# Mechanisms of membrane protein crystallization in ‘bicelles’

**DOI:** 10.1038/s41598-022-13945-0

**Published:** 2022-06-30

**Authors:** Tatiana N. Murugova, Oleksandr I. Ivankov, Yury L. Ryzhykau, Dmytro V. Soloviov, Kirill V. Kovalev, Daria V. Skachkova, Adam Round, Christian Baeken, Andrii V. Ishchenko, Oleksandr A. Volkov, Andrey V. Rogachev, Alexey V. Vlasov, Alexander I. Kuklin, Valentin I. Gordeliy

**Affiliations:** 1 Frank Laboratory of Neutron Physics, Joint Institute for Nuclear Research, 141980 Dubna, Russia; 2 Research Center for Mechanisms of Aging and Age-Related Diseases, Moscow Institute of Physics and Technology, 141700 Dolgoprudny, Russia; 3 Taras Shevchenko National University, Kyiv, 01033 Ukraine; 4 Institute for Safety Problems of Nuclear Power Plants of the Ukrainian NAS, Kyiv, 03028 Ukraine; 5 EMBL Hamburg Outstation, 22607 Hamburg, Germany; 6 Previously at EMBL-Grenoble Outstation, 38000 Grenoble, France; 7 Single Particles, Clusters, and Biomolecules and Serial Femtosecond Crystallography (SPB/SFX) Instrument, European XFEL GmbH, 22869 Schenefeld, Germany; 8 Institute of Biological Information Processing (IBI-7: Structural Biochemistry), Forschungszentrum Jülich, 52425 Jülich, Germany; 9 JuStruct: Jülich Center for Structural Biology, Forschungszentrum Jülich, 52428 Jülich, Germany; 10 Institut de Biologie Structurale Jean-Pierre Ebel, Université Grenoble Alpes–Commissariat à l’Energie Atomique et aux Energies Alternatives–CNRS, 38027 Grenoble, France

**Keywords:** SAXS, X-ray crystallography

## Abstract

Despite remarkable progress, mainly due to the development of LCP and ‘bicelle’ crystallization, lack of structural information remains a bottleneck in membrane protein (MP) research. A major reason is the absence of complete understanding of the mechanism of crystallization. Here we present small-angle scattering studies of the evolution of the “bicelle” crystallization matrix in the course of MP crystal growth. Initially, the matrix corresponds to liquid-like bicelle state. However, after adding the precipitant, the crystallization matrix transforms to jelly-like state. The data suggest that this final phase is composed of interconnected ribbon-like bilayers, where crystals grow. A small amount of multilamellar phase appears, and its volume increases concomitantly with the volume of growing crystals. We suggest that the lamellar phase surrounds the crystals and is critical for crystal growth, which is also common for LCP crystallization. The study discloses mechanisms of “bicelle” MP crystallization and will support rational design of crystallization.

## Introduction

Membrane proteins (MPs) play an essential role in living cell processes such as ion transport across the membrane, energy conversion, and signal transduction^1,2^. One-third of the human genome encodes membrane proteins. Due to their significant role in human physiology, membrane proteins are the targets of about 60% of currently used drugs^3,4^. To date, the most widely used method for obtaining high-resolution protein structures is X-ray crystallography, which requires high-quality protein crystals. However, the crystallization of membrane proteins remains a major challenge. Unique structures of membrane proteins^5^ account for only ~ 1% of all available unique high-resolution protein structures^6^. One of the *in meso* methods is crystallization in a bicellar mixture^7–11^. Nevertheless, this method leads to the elucidation of several important MPs, whose mechanism is still unclear and only relies on exhaustive trials and errors. The term “crystallization from bicelles” may relate only to the initial state of the matrix, and the evolution of the bicelle state to a matrix capable of supporting crystal growth has not been elucidated.

Considerable efforts have been taken to develop new methods, materials, and tools that could help overcome these stumbling blocks. However, despite past attempts, the rate of membrane protein structures deposition (the first membrane protein structure was deposited in 1985) is still far from those achieved for soluble proteins.

To overcome the challenge, a new method, i.e., MP crystallization in the lipid cubic phase (LCP) matrix, was introduced in 1996^12^. This approach allowed crystallization of challenging MPs (e.g., rhodopsins^13–15^ and G protein-coupled receptors) that have resisted crystallization with the standard vapor diffusion methods for decades^16–18^.

The *in meso* crystallization approach was further developed and expanded with other methods and tools, e.g., the utilization of lipids with varied properties to create the LCP matrix^19^, crystallization from MSP-based^20^ or polymer-bounded^21,22^ nanodiscs, and crystallization from bicelles^7,9,10^.

Earlier, bicelles were introduced as a membrane mimicking model potentially superior to micelles^23^. It was shown that some mixtures of lipids and detergents form disc-shaped particles, with a lipidic bilayer, a core, and a detergent-stabilized rim providing a stabilizing environment for MPs by mimicking native cellular membranes. Dimyristoyl phosphatidylcholine (DMPC) is often used as a long-chain phospholipid component of bicelles, and it can be doped with phospholipids with similar chain lengths but different head groups. On the other hand, the rim can be stabilized by a bile-salt short-chain derivative such as 3-(cholamidopropyl) dimethylammonio-2-hydroxy-1-propanesulfonate (CHAPSO). Since the long-chain phospholipid in bicelles is sequestered into the planar core region, which is devoid of short-chain phospholipid or detergent, the core region of a bicelle mimics a section of natural membrane much better than conventional detergent micelles. The size and the properties of these lipid particles can be varied as a function of the ratio of a long-chain lipid to a detergent or a short-chain lipid (Q-ratio) and lipid concentration. The variation of Q provides a variety of similar bicelle phases that are amenable to different types of biological studies. In general, at a high lipid ratio (Q > 2) and a wide concentration range (C_lp_ = 0.25–25% (w/w)), bicelles with a diameter of approximately 100–500 Å are formed^24–33^. The decrease of Q results in smaller bicelles^24,34–36^.

Bicellar mixtures show structural plasticity depending on the lipid to detergent (or a short-chain lipid) ratio (Q), lipid concentration (C_lp_), temperature (T), and chemical composition^24,25,28,29,31,37–45^.

In 2002, for the first time, S. Faham and J.U. Bowie presented a new method for crystallizing membrane proteins based on bicelle forming lipid/detergent systems^7^. The bacteriorhodopsin (BR) crystals obtained with this approach belonged to the space group P2_1_ with unit cell dimensions of *a* = 45.0 Å, *b* = 108.9 Å, *c* = 55.9 Å, *β* = 113.58°, and a dimeric asymmetric unit and diffracted to high resolution. It is considered that the crystals of BR were of type 1 with a typical membrane-like sandwich packing of proteins, as it is in the case of all crystals grown by the LCP approach. Since that time, several important membrane proteins have been crystallized by this approach^7,46–72^ (see Table 1 and its’ extended version Table S1).

**Table 1:** The list of the membrane proteins whose structure was resolved by X-ray crystallography using bicellar systems.

Protein	PDB ID	Crystal type	Ref
Bacteriorhodopsin	1KME	1	^7^
Bacteriorhodopsin	1XJI	1	^46^
Bacteriorhodopsin	4HYX	1	^47^
Proteorhodopsin	4JQ6	1	^48^
β2-Adrenergic receptor	2R4S, 2R4R	1	^49^
Voltage-dependent anion channel 1	3EMN	1	^50^
Voltage-dependent anion channel 1	5XDN	1	^51^
Voltage-gated sodium channel	3RW0, 3RVY, 3RVZ	1	^52^
Ca2 + selectivity of a voltage-gated calcium channel	4MS2, 4MTO, and others	1	^53^
Efonidipine-CavAb complex calcium-selective channel	6JUH	2	^54^
Xanthorhodopsin	3DDL	1	^55^
Rhomboid protease	2XTV, 2XTU	1	^56^
EIIAGlc in complex with the maltose transporter, an ATP-binding cassette (ABC) transporter	4JBW	2	^57^
TamA	4C00	1	^58^
LeuT (prokaryotic ortholog of the NSS family)	3USI and others	1	^59^
SaMGT (transglycosylase)	3VMT	1	^60^
Cellulose synthase	4P02	2	^61^
C10 ring of ATP synthase	3U2F	1	^62^
N-terminally truncated BamA (HdBamAΔ3)	4K3C	1	^63^
Full-length BamA construct from N.gonorrhoeae (NgBamA)	4K3B	1	^63^
mVDAC1	4C69	1	^64^
Rhomboid protease GlpG	5F5D, 5F5G, 5F5J, 5F5K	1	^65^
Gloeobacter rhodopsin	6NWD	1	^66^
Human sterol transporter ABCG5/ABCG8 (ATP-binding cassette sub-family G member 5)	5DO7	2	^67^
ABC-type bacteriocin transporter PCAT1	4RY2	1	^68^
Membrane domain of respiratory complex I from *E. coli*	3M9C	1	^69^
Membrane domain of respiratory complex I from *E. coli*	3RKO	1	^70^
Murine voltage-dependent anion channel	7KUH	1	^71^
Chloride-pumping rhodopsin MastR	6XL3, 6WP8	1	^72^

The additional information such as bicelle formulation, temperature conditions, buffer composition and parameters of a soluble part of proteins is provided in Supplementary Table S1.

Contrary to the dominating paradigm, our analysis of the literature data shows that the published structures obtained with the crystals grown with the “bicelle” approach were of both types (not only type I crystals typical of *in meso* crystallization). While integral proteins without any water-soluble domains usually form layer-like type I crystals, proteins with relatively large (50 Å and more) water-soluble regions tend to form type II crystals. However, type I crystals can form even in the latter case depending on crystallization conditions (see Table S1). Notably, there is no certain dependence of the crystal type on the composition of the precipitant solution. It may mean that the membrane protein crystal growing from the initial bicelle state may go through principally different routes of the structural state of the crystallization matrix resulting in two principally different types of crystals. The main focus of this work is the growth of type I crystals.

The morphology of bicelle systems has been investigated by SAS, NMR, AFM, and EM^25,28,29,31,37–43,73^. For Q suitable for crystallization (around Q = 3), the summarized phase diagram has specific regions, including bicelles, nematic phases, such as branched worm-like micelles or ribbon-like structures, multilamellar structures, and perforated membranes (see Supplementary Figure S1(A)). It is known that the phase state of lipidic systems depends on temperature, which may impact protein crystallization^74^.

Interestingly, although the dependence of the system on temperature is complex, most of the crystals grown with the bicelles method were obtained at room temperature. In general, at low temperatures and lipid concentrations, the system is a liquid suspension containing bicelles. As temperature and concentration C_lp_ increase, the system forms worm- and ribbon-like structures^28,30,37–39,75,76^. A further increase in temperature or concentration causes a transition into a gel phase consisting of unilamellar or multilamellar structures and perforated membranes^24,28,29,37–39,75,77,78^. A similar system of DMPC/CHAPSO to the one used in this study was described previously^24^. In a temperature range from room temperature up to 32 °C, this system is comprised of bicellar and ribbon-like structures, whereas a part of the diagram was not defined (Figure S1(B)).

Most of the published phase diagrams were obtained for pure aqueous suspensions of the bicelles. In the case of a crystallization experiment, a precipitant and an MP are added to the system, and its morphology can change. It has been shown that the phase behavior of the bicellar mixture is affected by a membrane charge and buffer salinity. For instance, high salinity promotes vesiculation and the formation of aggregates in charged DMPC/DHPC/DGPC systems^79^. In turn, the presence of a charge on the membrane can induce perforation in the lipid bilayer^24,30,80^.

Thus the often used term “crystallization from bicelles” may only mean that the starting crystallization matrix is a liquid phase comprising of bicelles, membrane proteins (surrounded by native membranes or membrane mimicking systems) and buffer. However, what happens with the crystallization matrix after the initiation of crystallization (upon adding precipitant) and what the phase state (structure) is when crystals grow is not known.

Here, we present the results of the small-angle X-ray and neutron scattering (SAXS and SANS) studies of structural evolution of the crystallization matrix from the initial bicelle to the final jelly-like state where MP crystals grow. At low Q values, amphiphiles form micelles^81^, whereas, as Q increases, they tend to form bicelles. For protein crystallization, the mixtures with Q = 2–3 are used, and the most frequently utilized amphiphiles are DMPC and CHAPSO (or DHPC) (see the list in Table S1)^7,46–70^.

The growth of the membrane protein crystals was monitored simultaneously in the same experiments. The final (jelly-like) state of the matrix, where the crystals were growing, was formed by interconnected ribbon-like bilayers.

## Results

### The scheme of the experiment

**Figure 1:**
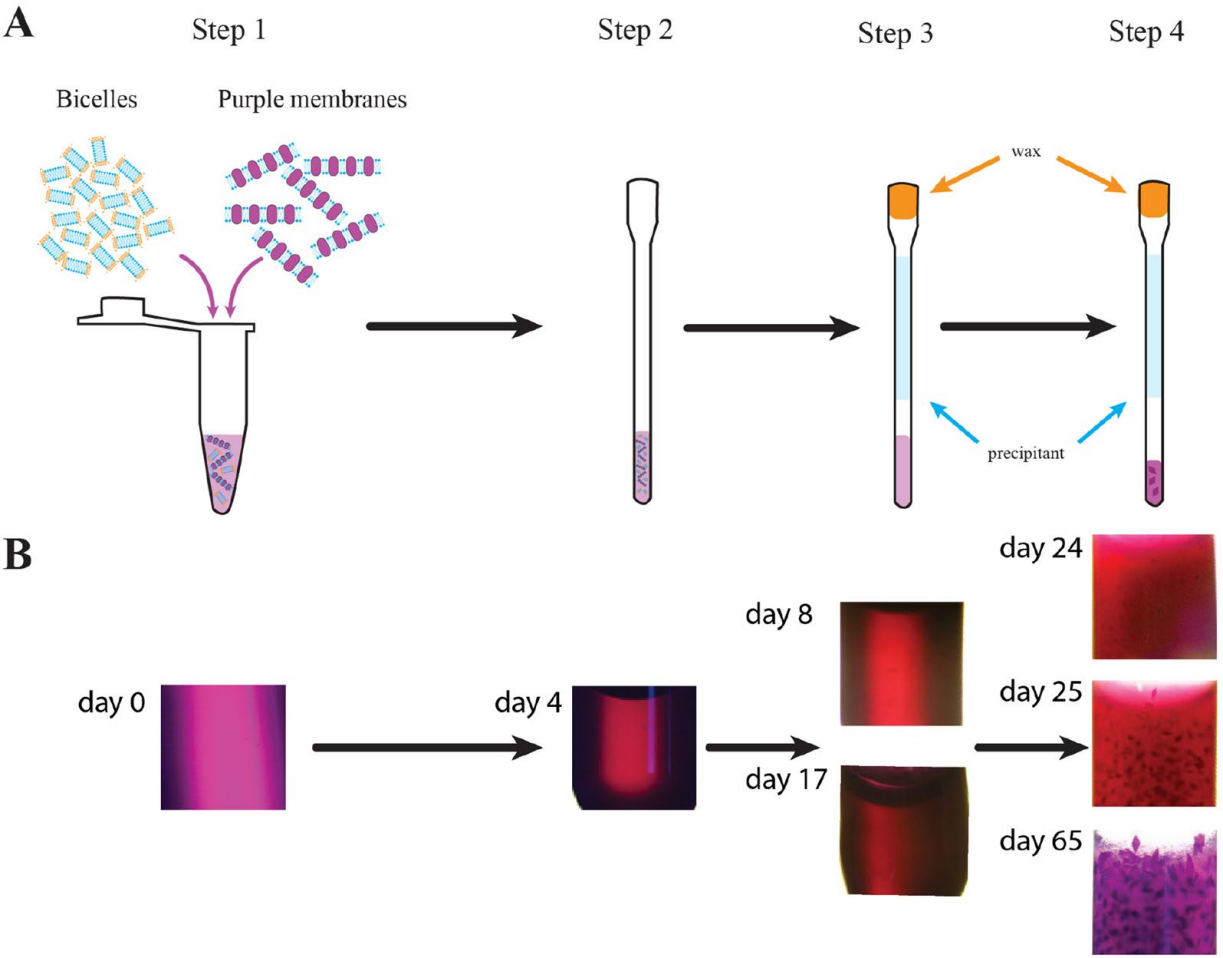
Sequence of the experimental steps. (**A**) Schematic representation of the experimental stages of crystallization of bacteriorhodopsin in bicelles within capillaries. Step 1—preparation of the crystallization system on ice (mixture of bicelles and purple membranes); step 2—loading of the bicellar/purple membrane mixture into a capillary; step 3—addition of a precipitant and monitoring of subsequent changes of the crystallization matrix; step 4—monitoring of the formation of the BR crystals. During steps 2–4, monitoring of the structure of the crystallization system and the growth of the crystals were performed using real-time SAXS. (**B**)—Photographs of the crystallization system in different steps of the experiments (aligned vertically with **A**) corresponding to steps 1 (day 0), 2 (day 4), 3 (day 8 & 17); the crystallization system presents a transparent/semi-transparent homogeneous phase. In step 4 (day 24, 25 & 65), small crystals appeared, and then they grew to a maximum size.

For our study, we used a well-tested crystallization system and conditions where the formation of BR crystals was previously observed^7,46^. The experiments were divided into the following four steps (Fig. 1A): (1) preparation of the crystallization system on ice; (2) loading of the crystallization system into a glass capillary; (3) addition of a precipitant and real-time monitoring of the structure evolution of the crystallization matrix; (4) real-time monitoring of the formation of bacteriorhodopsin crystals in the crystallization matrix.

**Figure 2:**
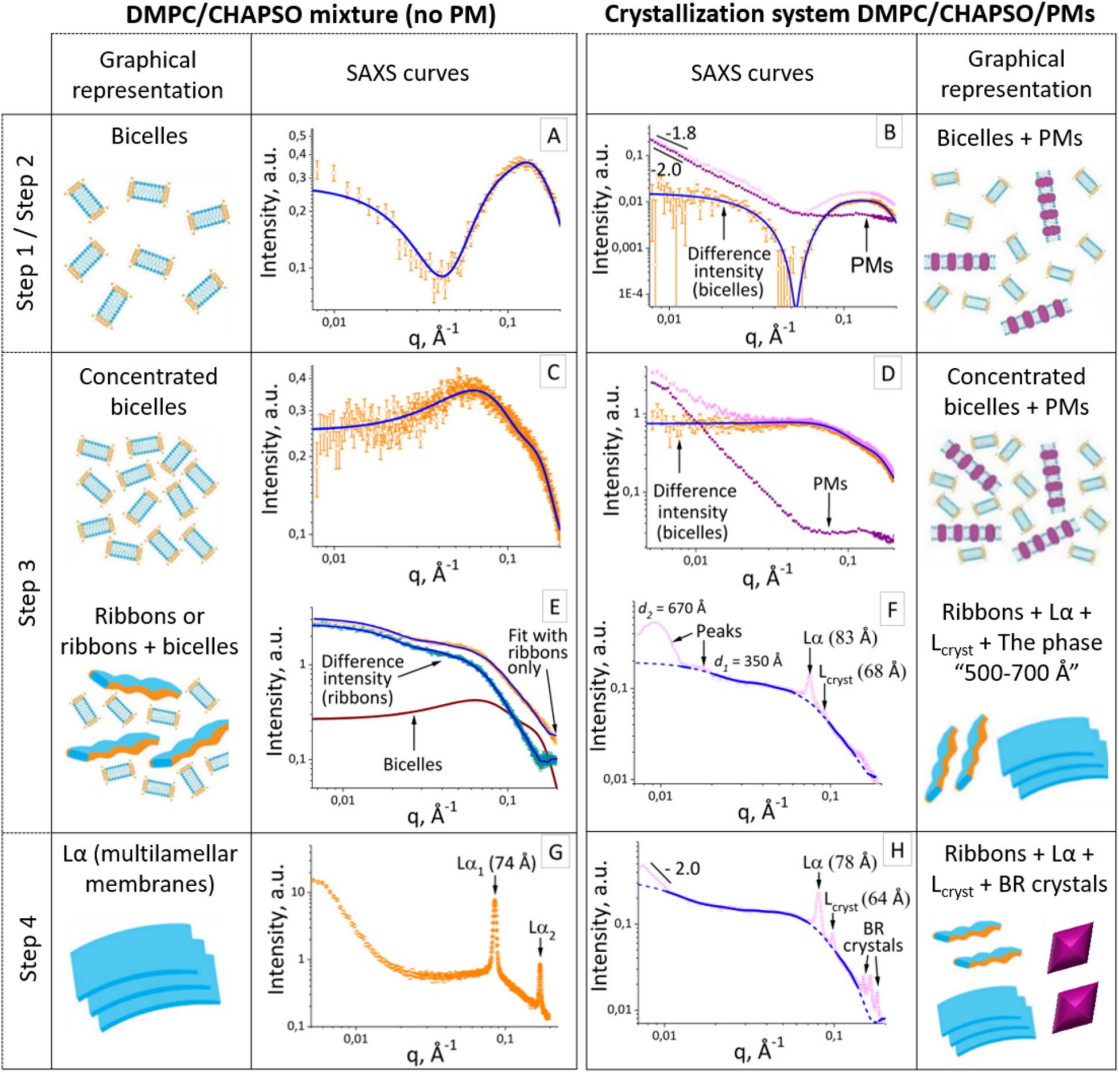
Evolution of the crystallization matrix during the crystallization process. The SAXS curves for the pure DMPC/CHAPSO mixture without PM (left) and the crystallization system DMPC/CHAPSO/PM (right). Experimental data for crystallization system with and without PM are shown as light purple and orange hollow circles, correspondingly. SAXS curves for PM are shown as dark purple squares. The approximations by form-factors of the bicelles and the ribbons are shown as blue lines. The graphical representations of the structural organizations of the crystallization matrix are shown adjacent to the corresponding SAXS curves.

**Figure 3:**
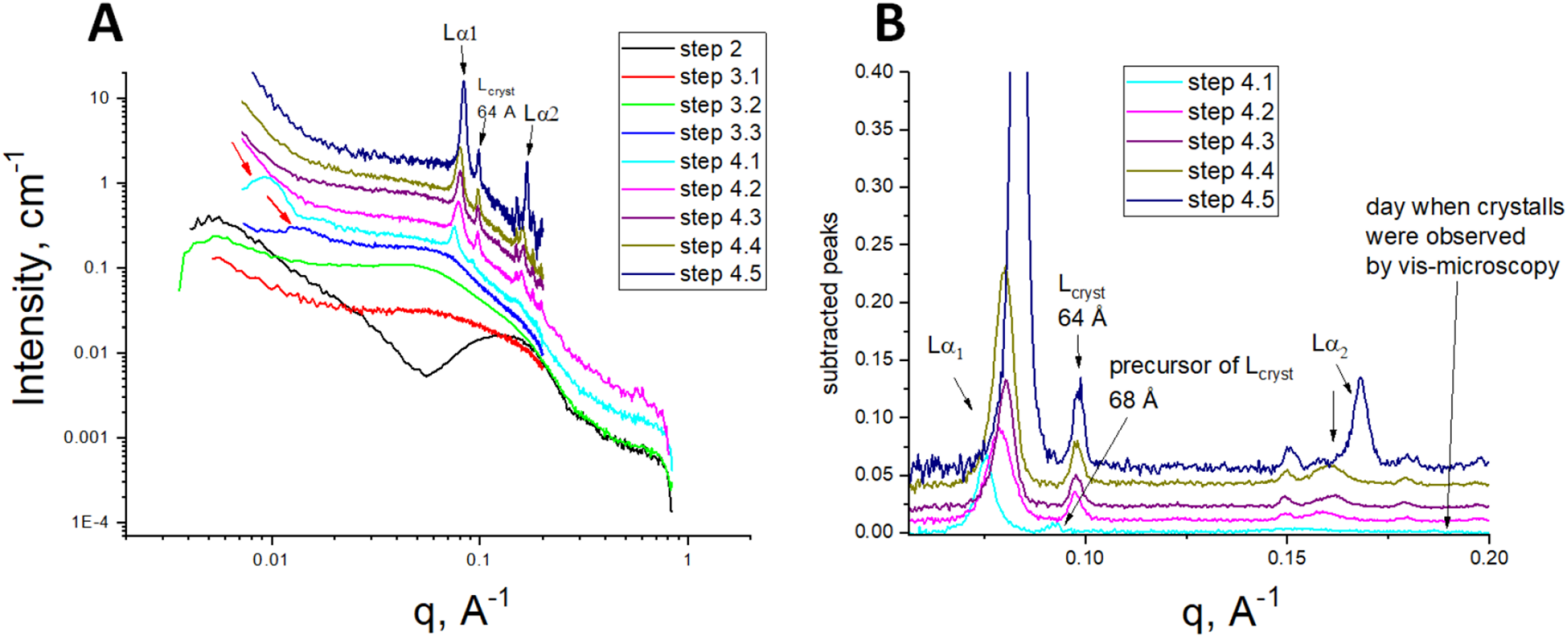
Transformation of the SAXS curves for the crystallization system during the different steps of the crystallization process. (**A**) The SAXS curves related to different steps of the crystallization process. The red arrows in the small angle region indicate the interference peaks from the lipid/detergent smectic phase (curve designations are given in the legend; steps numbering is described in Fig. 1). (**B**) The peaks extracted from the SAXS curves (part **A**) by subtraction of the baseline. The curves are scaled to separate them vertically for better visualization. The black arrows “Lα_1_” and “Lα_2_” (shown in both **A**,**B**) indicate the lamellar peaks of the first and second order; the other black arrow “L_cryst_ 64 Å” indicates the peak from the local lamellar phase bonded with the protein surface, the spacing of this L_cryst_ is 64 Å. The arrow “68 Å” indicates the precursor of the L_cryst_ phase.

During steps 2–4, the monitoring of the crystallization system and the growth of crystals were performed using SAXS (Figs. 2, 3). In parallel, the system was observed using an optical microscope (Olympus SZX-ILLK200) as well (Fig. 1B).

In addition to the crystallization system containing protein, we also studied (as a reference) the same system under the same conditions, but without protein. The data and the calculations indicate that the presence of protein does not affect the morphology of the crystallization matrix. The calculated ratio of BR to DMPC in our system (the molar ratio 1:200; the cross-section area ratio in a membrane 1:17; the volume 1:6) supports our conclusions.

### Step 1. Preparation of the crystallization system

Preparation of the crystallization system (protein/bicelle mixture) was performed on ice according to the standard procedure described in the “Materials and Methods” section. It is worth noting that instead of the detergent-solubilized BR, we used purple membranes. The system was assumed to present a bicellar structure since the mixture DMPC/CHAPSO forms bicelles at low concentrations and low temperatures^46^. To verify this, we carried out all our SAXS experiments at room temperature; between the SAXS measurements, the capillaries were kept in a temperature-controlled box at 32 °C. The initial concentration of the DMPC/CHAPSO mixture was 5.6%, which is low enough for the bicelles at room temperature as well. Using the scattering method, we observed the formation of the bicelles both in our pure system (no protein) in an appropriate buffer (Fig. 2A) and in the crystallization system in step 1, step 2 (Fig. 2B).

### Step 2. Loading the sample into capillary

#### Initial state of the crystallization system

The cooled crystallization system was transferred into the capillary (see “Materials and Methods” and Figure S2). Then the crystallization system was studied by SAXS at room temperature. For comparison, the purple membrane suspension was loaded into another capillary. This suspension contained the same protein concentration as the crystallization system (8.4 mg/ml). The scattering curves for both samples are presented in Fig. 2B. In the small-angle region (up to 0.04 Å^−1^), the curves follow a linear behavior with a slope of −1.8 for the crystallization system and −2.0 for the purple membranes. These slopes indicate large lamellar structures in the samples, in our case, the purple membranes. We subtracted the scattering intensity from the purple membranes from the intensity for the crystallization system. The result is shown in Fig. 2B (the curve is marked as “difference intensity”). The difference intensity can be fitted by a form-factor for the bicelles (Model 1) with a radius of 50 Å and a height of 45 Å (see fit in Fig. 2B and fit parameters in Table S2). In addition, we examine the structure of the pure bicellar crystallization matrix in the absence of purple membranes using SAXS and SANS (see “Materials and Methods” section for details). The crystallization system presents a bicellar mixture with similar parameters described in Table S2, and the fitting curves are presented in Fig. 2A (for the SAXS experiment) and Figure S3 (for SANS). The SAXS and SANS data for these systems are consistent. For fitting the SAXS/SANS data from the bicelles, we used Model 1 of a cylinder with a core–shell scattering length density (SLD) profile, which is described in “Materials and Methods”. Similar model was successfully used in the previous SAS studies of bicelles with different lipid/detergent contents^44,82–84^ (see also Section “SAXS profiles for bicelles” in Text Document S2).

On average, the core radii are about 40 Å; considering the belt thickness results in the total bicelle diameter of ~ 100 Å (see Section “Differences in bicelle radii” in Text Document S2). The diameter of the bicelles observed in work^24^ for the analogical system (DMPC/CHAPSO mixture, Q = 3) and obtained using electron microscopy was in a range of 100–500 Å. In accordance with their SANS model fit, the diameter was about 420 Å. However, in the mentioned work, the total lipid/detergent concentration was 0.25 wt %, whereas, in our experiments, it was in a range from 5.6 to 14%. As shown in^38^, the average size of bicelles critically depends on the total lipid/detergent concentration: the pseudo-hydrodynamic radius decreases by several times when the total lipid concentration increases by several times. Therefore, the bicelle diameter of ~ 100 Å obtained in our study aligns with the current data on bicelles.

The datasets corresponding to the mixture of the bicelles with PMs (i.e., the crystallization system) at steps 1–2 could be described as a sum of the scattering data from PMs and from pure bicelles (see Fig. 2B and Section “Fitting of difference intensities” in Text Document S2). Thus, we can describe the crystallization system before adding a precipitant as a simple mixture of bicelles and purple membranes.

### Step 3. Addition of a precipitant

#### Evolution of crystallization matrix before crystals nucleation

After the crystallization system (purple membrane/bicelle mixture) was loaded to the capillary, a precipitant was added above the crystallization mixture to the same capillary with an air gap between the crystallization system and the precipitant (see “Experimental” section). From this moment, the volume of the crystallization system began to shrink due to the diffusion of water from the crystallization matrix into the precipitant solution. After a week, the volume decreased by 50–60%, and the next week, it corresponded to 40% of the initial volume. Afterward, only minor changes were observed. The diagram of the volume evaluation decrease is presented in Figure S4.

The evolution of the SAXS curves during step 3 is presented in Fig. 3. The curves underwent significant changes, and the crystallization matrix took a transient state. Then the matrix became stabilized and did not change its morphology. This last state is a focus of our thorough study as it shows that the crystals start to grow at this state of the crystallization matrix.

It is important to note that the crucial changes in the SAXS profile at early stages of Step 3 (Fig. 2C,D) are mainly caused by an increase in salt concentration. A buffer with increased salt concentration has a higher electron density (see Section “Buffer SLD values at different steps” in Text Document S2), which is why the ratio between the contrasts *Δρ* = *ρ* – *ρ*_*buffer*_ of the bicelle components dramatically changes with salt concentration, leading to a variation of the SAXS signal. 

At this stage, as in step 2, the crystallization system is a simple mixture of bicelles and purple membranes (see Fig. 2D and Section “Fitting of difference intensities” in Text Document S2), but their concentration is higher due to evaporation the water from the crystallization system. Meanwhile, structural parameters of bicelles undergo slight changes (see Table S2 and Section “*SLD changes in bicelles*” in Text Document S2).

#### Formation of ribbons

With continued drying, the system undergoes morphological changes. For the interpretation of SAXS curve in the later stage of the step 3 (Fig. 2E,F), we were guided by the phase diagram obtained for the DMPC/CHAPSO mixture in^24^, where the molar ratio Q = 3 was close to Q = 2.7 in our experiments. The temperature and the concentration conditions in our experiments are marked by a purple rectangle on the diagram presented in Figure S1. Previously, the reorganization of bicelles into elongated ribbon- and worm-like aggregates was detected by different methods (SAS, electron microscopy, NMR, polarized optical microscopy)^24,38,75,85,86^. Since the DMPC tends to form a bilayer, these elongated “worm-like” objects should have a flattened cross-section (ribbon-like) with a thickness close to the thickness of the DMPC bilayer. Thus for the approximation of SAXS on the ribbons, the model of an elliptical cylinder with a core–shell scattering length density profile was used (see “Materials and Methods”, Model 2). The analogous model of the elliptical cylinder was already used in^24^ for fitting of the SANS data; however, in the mentioned work, the cylinder was assumed to be of equal density, which is sufficient to fit the SANS data. The case of the SAXS data requires a core–shell model for theoretical approximations because the hydrophobic and hydrophilic parts of micelles and/or bilayers have a different sign of contrast *Δρ* = *ρ* – *ρ*_*buffer*_ (see, for example, Table S3). In fact, the total ribbon thickness was observed to be slightly larger than the expected thickness of the DMPC bilayer both in our calculations (see Table S3) and in the work^24^.

The formation of ribbons from bicelles is associated with the DMPC redistribution and the CHAPSO molecules between the head and the belt region of the bicells, correspondingly resulting in the SLD unification and the thickness of the hydrophilic shell of the ribbon. Therefore, the thickness of the resulting hydrophilic shell of the ribbon is fixed on the value *T*_*shell*_ = *Max* *(H*_*head*_*,* *ΔR)* = *ΔR* = 11.4 Å.

As ribbons originate from the initial bicelle phase, we expect the existence of intermediate phases presented by bicelles and ribbons. This assumption is in agreement with the corresponding regions of the phase diagram of the bicelles (see Figure S1B). Thus we observed the coexistence of bicelles and ribbons in pure DMPC/CHAPSO mixture (without PMs). The scattering curve for this system is approximated with two models: a ribbon and a mixture of the ribbon with bicelles. The results of both approaches are shown in Table S3 and Fig. 2E. In general, the obtained structural parameters do not differ dramatically: ribbon’s length (L) is about 330 Å in both cases, minor/major radii of hydrophobic core of ribbons are 25 Å/47 Å for the case of ribbon/bicelle mixture and 20 Å/43 Å for ribbons only (see Table S3). However, in the case of the ribbon/bicelle mixture, the χ^2^ value is much lower (1.2 instead of 3.5) and corresponds to more precise approximation.

In accordance with the volumes of the hydrophobic cores of the ribbons and the bicelles in the case of the DMPC/CHAPSO mixture (without PM), at the stage of the formation of the ribbons, for which the scattering curve in Fig. 2E was obtained, one ribbon is formed from approximately ten bicelles.

SAXS curve (see Fig. 2F) obtained at the last stages of step 3 for the crystallization system (DMPC/CHAPSO/PMs) contains diffraction peaks from the lamellar phases Lα, L_cryst_ and the “phase 500–700 Å”. In general, the background curve here is similar to the scattering curve from ribbons in case of the DMPC/CHAPSO mixture (see Fig. 2E). In particular, the background is well approximated with the form-factor of ribbons (see solid line in Fig. 2F), and obtained structural parameters are close to those obtained for ribbons in “pure” DMPC/CHAPSO mixture (see Table S3). Therefore, the ribbons are the main component of the crystallization system at this stage of the crystallization process.

Importantly, the scattering curve at this stage does not follow a linear behavior with a slope of −2.0 typical for oblate structures. This might imply that PMs in their initial state are absent at this stage. At least, the concentration of the remaining PMs is not enough to be detected by SAXS. It indicates that a majority of PMs dissociates synchronically with the transition from bicelles to ribbons, and BR molecules incorporated directly into ribbons. Considering that the amount of purple membranes is an order of magnitude less than the DMPS/CHAPSO mixture, we assume that all purple membranes should dissolve.

#### Formation of phase 500–700 Å

In the later steps of crystallization, the matrix stabilized, and the SAXS curves did not show any further significant changes. At this stage several peaks are observed in the SAXS curves (Figs. 2F and 3). There are two groups of peaks of different origins. We give them following designations: 500–700 Å and Lα. The first group presents wide peaks in a small angle region *q* < 0.025 Å^−1^ (in Fig. 3A, the curves indicated as steps 3.3 and 4.1; red arrows indicate the peaks). These peaks were observed from one to seven days before and one day after crystals were detected. Then these peaks disappeared as the crystals started growing. These peaks were accompanied by the other group of the lamellar peaks Lα for 70–80 Å (will be described in the next subsection) or were observed just before appearing of these Lα lamellar peaks (see Step 3.3 in Fig. 3A).

The first peak position corresponds to the lattice parameter *d* = 2π / *q*_*max*_ 500–700 Å (average value for series of samples is 660 Å). The second peak corresponding to *d* = 2π / *q*_*max*_ ~ 350 Å could be the second-order peak of the (660 Å) peak mentioned above; the peak position ratio varies from 1:1.79 to 1:1.89. Or these two peaks can have different origins since the ratio of the peaks’ positions is not equal to 1:2. It is worth noting that their appearance is synchronized (see the “Discussion” section), and the lattice parameter *d* ~ 350 Å may be very close to the ribbon length. Unfortunately, we cannot make conclusions on the origin of this peak since the length of the ribbons cannot be estimated accurately due to the unavailability of the data in the q-range corresponding to the condition *q L* ≪ 1.

**Figure 4:**
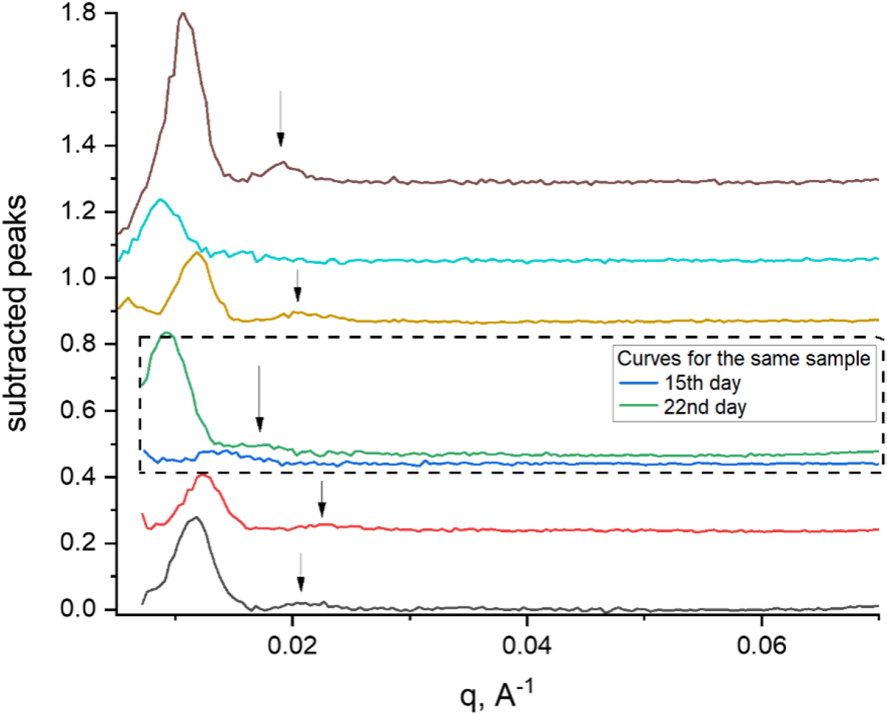
SAXS peaks from the lipid/detergent crystallization phase prior to or at the moment of crystal formation. The peaks’ positions correspond to distances 500–700 Å (these values were calculated from the positions of 1st order peaks). The graphs are presented after baseline subtraction. The observed peaks disappeared several days after they appeared. Each curve is measured in a different capillary. The curves are scaled to separate them vertically for better visualization. The dashed rectangle highlights two consecutive curves of the same sample: these curves show that, with time, peak intensity increases and shifts to smaller angles. The arrows indicate the position of the 2nd order peaks.

Since the small angle peaks are transient, it was challenging to monitor their behavior with the same sample. We have only two consecutive curves indicating that the peak increased in its intensity, and its maximum shifted into smaller angles (highlighted by the dashed rectangle in Fig. 4). Our discussion of the possible nature of these peaks is given in the “Discussion” section.

#### Formation of the lamellar phase Lα

The second group of peaks corresponds to the lamellar phase Lα with the lattice parameter *d* = 70–80 Å. There are two lamellar diffraction peaks in the middle range of the scattering vectors *q* (Fig. 3, indicated as “Lα_1_” and “Lα_2_”). The ratio between the peaks’ maximum positions is 1:2. These peaks appeared before the BR crystals formation and remained in the curves even after the formation of the crystals during our entire observation period (two months). Their intensity increased, and the position of maximum shifted to greater *q* during the time (Fig. 3B and Figure S5). In addition, these peaks were observed in the curves for the samples where we did not find BR crystals. We studied the structural behavior of the pure lipidic matrix in the absence of BR under crystallization conditions and discovered that the lamellar peaks were observed there as well in the last step of the lipidic matrix drying (Fig. 2G), which indicates that the nature of these peaks originates from the lipidic matrix.

We estimated the lamellar distances *d* from the 1st peak position *q*_*m*1_ as *d* = 2π / *q*_*m*1_. First, when the lamellar peaks appeared, they corresponded to the distance of about 84 Å, and then, with time, to 73 Å. These values were summarized for a set of samples (13 items). The repeat distance *d* for the lipid matrix without BR corresponds to 74 Å. The transition of the bicellar mixture into the lamellar phase was reported for the pure DMPC/CHAPSO and DMPC/DHPC systems at increasing temperatures^24,25,29,31,73,87^. The reported distances for the pure DMPC/CHAPSO and DMPC/DHPC mixtures are about 62 Å and 65 Å accordingly^24,29^, and for the multilamellar vesicles of the pure DMPC are around 62 Å^44,88^. The greater value of 73 Å in our experiments can be caused by the presence of a high-concentrated buffer and BR molecules.

There is evidence that bicellar mixtures can form perforated membranes^24,37–39,75,80,89,90^, where pores are rimmed by a short-chain detergent. SAXS is unable to distinguish a homogeneous bilayer from a perforated one. Nevertheless, we hypothesize that putative perforations should exist to connect lamellar membranes and help protein migrate from bilayers to the place of the crystal growth which is necessary conditions for the crystals growth. Further work to obtain evidence to support this hypothesis is planned.

Thus, after the addition of the precipitate, the crystallization system, which is initially a mixture of bicelles and purple membranes, begins to shrink: the concentration of bicelles and purple membranes increases. Then the bicelles fuse into ribbons, this process is accompanied by the dissolution of purple membranes. Also at this step, the formation of a temporary phase with characteristic parameters of 500–600 Å is observed. A multilamellar phase is also formed with an average lattice parameters of 73 Å. This phase remains at the Step 4, when crystal growth is observed.

### Step 4. Growth of crystals

When the crystallization matrix was stabilized, the BR crystals started to grow. We observed the appearance of the crystals by a vis-microscope (see “Materials and methods”) in the samples stored at 32 °C from two to three weeks after the capillary filling, and in five weeks, in the samples stored during 4 weeks at room temperature and then moved to a temperature-controlled box with a temperature of 32 °C. Notably, the movement of the samples to 32 °C speeded up the appearance of the crystals. The crystal growth was accompanied by diffraction peaks appearing in the scattering curves in the wide-angle range (Fig. 3, steps number 4) corresponding to the crystal lattice (see below). The intensities of the crystal peaks increased as the protein crystals grew (see Figure S5). These crystal peaks were always observed in the presence of the lamellar phase peaks Lα_1_, Lα_2_ as mentioned above in a section “Step 3”.

#### Identification of the diffraction peaks and the parameters of the BR crystals

**Figure 5:**
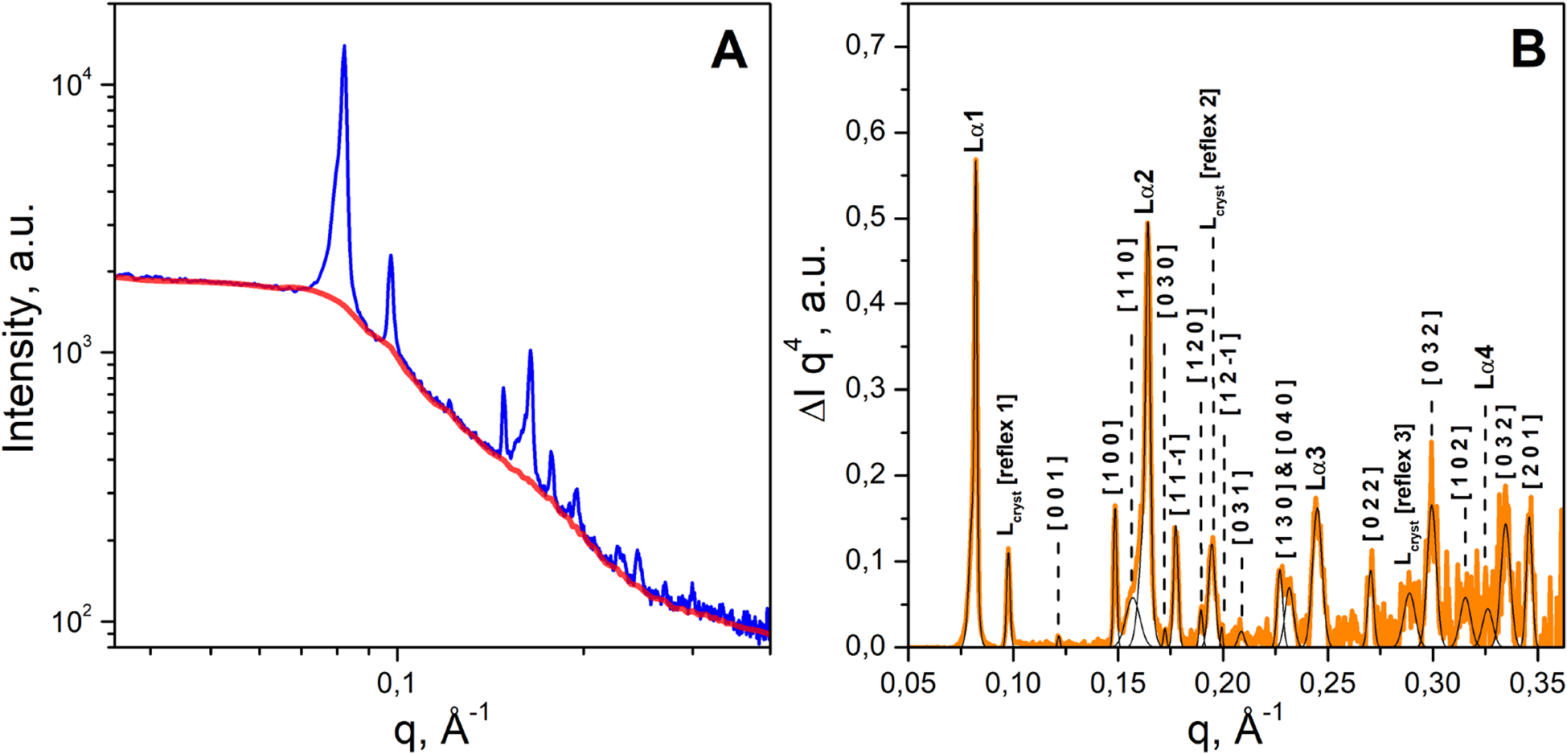
SAXS data from the crystallization matrix after crystal formation (BM29, ESRF). (**A**) The SAXS curve for the crystallization matrix with crystals (blue curve) and the corresponding baseline (red curve). (**B**) The SAXS curve from the crystallization matrix after baseline subtraction (orange). Intensity is multiplied by q^4^ for better observation of the wide-angle peaks. The Gaussian approximations of the peaks are represented in black. The Miller indexes (for BR crystals) and the reflex numbers (for the lipidic multilayers Lα or L_cryst_) are marked above the corresponding peaks (for more details, see Table S4).

Bacteriorhodopsin crystals belonging to the space group P2_1_ with unit cell dimensions of *a* = 45.0 Å, *b* = 108.9 Å, *c* = 55.9 Å, *β* = 113.58° were obtained by a method of crystallizing membrane proteins in ´bicelle´ systems in the previously reported work^7^. In our crystallization experiments, the crystals with the same space group were found. Using the peak positions (see Table S4) obtained from the SAXS data (see initial and subtracted data in Fig. 5A,B, correspondingly) for the crystallization matrix after crystal formation, we calculated the unit cell dimensions as *a* = 43.91 Å, *b* = 109.33 Å, *c* = 53.4 Å, *β* = 104.63°, which turned out to be close to the previously reported data^7^ (see details of calculation of unit-cell dimensions in “Materials and Methods”).

#### *Formation of the lamellar phase presumed to be associated with the crystal surface (L*_*cryst*_*)*

**Figure 6:**
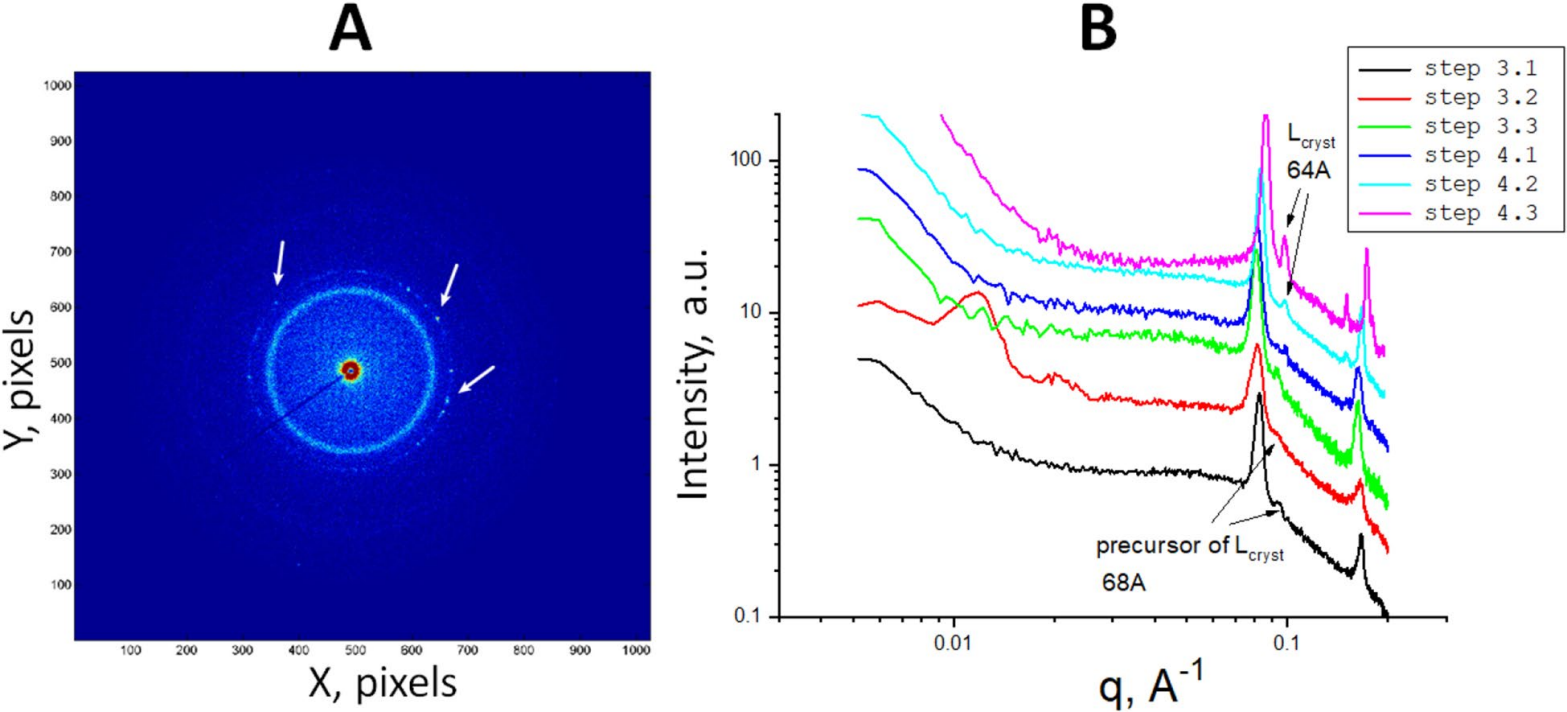
Behavior of the peak for the local lamellar phase L_cryst_. (**A**) 2D pattern for the sample containing the BR crystals (corresponds to 1D curve step 4.3 in Fig. 3). The reflections corresponding to the local lamellar phase L_cryst_ are shown by white arrows. The scattering ring belongs to the multilamellar phase Lα with a spacing of about 84 Å. (**B**) Behavior of the peak for the local lamellar phase L_cryst_ on the 1D curves. All the SAXS curves belong to different time points of the same sample. The crystallization conditions are the same as for the sample in Fig. 3.

There is a diffraction peak at 63.9 ± 0.4 Å (averaged value for seven samples, 21 curves) that we cannot associate with the BR crystals since this diffraction peak's position is beyond the parameters of the BR lattice spacing. On 2D SAXS patterns, this peak is presented as point reflexes (Fig. 6A). Thus we suppose that this peak can be attributed to a quasi-crystalline lipidic phase. This peak is always observed in the presence of lamellar Lα peaks and sometimes before the BR crystals can be observed by vis-microscopy and SAXS. Its amplitude changes concomitantly with the intensities of the BR crystals peaks (Figure S5). The positions of the L_cryst_ peak and the crystals’ peaks remain unchanged within their error bars during the observation time. The described peak can have a “precursor”: for several samples, we were able to register a peak at 68 Å (Figs. 3B and 6B), then after several days, it shifted to 64 Å and retained that position. The same position at 68 Å was observed for several samples in which we did not register the BR crystals either by vis-microscopy or SAXS. In this case, the peak position did not change during the observation time (60 days). The crystallization system in these cases contains unformed protein aggregates, as seen by vis-microscopy (see Figure S6(A, B)). We assume that there is lipid-protein nucleation at the beginning of crystal formation; this nucleus has a characteristic spacing 68 Å reflected in the appearance of a “precursor” peak. Then a crystal starts to grow with fixed lattice spacing, and the observed peak shifts from 68 Å to 64 Å and no longer changes this position during crystal growth. We suggest that the 64 Å peak can correspond to the local lateral lipidic phase physically bounded with a BR crystal. It is similar to the lamellar system described for the LCP crystallization^17,91–93^. Direct observation of the local lamellar phase in the case of crystallization in LCP is described for BR^93^ and transmembrane peptide DAP12-M^94^. Since this local lipid phase is physically attached to the crystal, it is expected to be oriented relative to the crystal. Moreover, the planes of the membranes are likely to be parallel to the planes of “type I” crystals. The orientation of this local phase implies that the “precursor” peak in the 2D scattering image is represented by a set of separate diffraction peaks, which can be observed in our data (see Fig. 6A).

Thus after equilibration of the crystallization system, the formation and growth of crystals begin. At this stage, ribbons are dominantly presented, which might help proteins to migrate to the centers of crystal formation from a multilamellar phase Lα with the lattice parameter 73 Å. We also observed the formation of the lamellar phase presumably connected directly with the crystal surface (L_cryst_) with the lattice parameter 64 Å. This phase might allow proteins to diffuse to the crystal surface.

## Discussion

Using SAXS, we investigated the structural evolution of the crystallization matrix during crystallization of bacteriorhodopsin in the DMPC/CHAPSO mixture, initially in a bicellar form. We showed that in the initial step, the matrix presents a mixture of bicelles and purple membranes. Thus, initially, the protein is not incorporated into the bicelles, in contrast to other crystallization systems, like LCP and vesicles, where protein solubilized in a detergent is added to LCP.

At the beginning of evaporation, SAXS curves demonstrate significant changes. The changes are caused by increased salt concentration and the corresponding increase of the electron density of the buffer. However, DMPC and CHAPSO are still assembled into bicelles.

In the next steps, in the presence of a precipitant and evaporation water from the matrix, the latter transforms into a phase made up of ribbon-like structures. In this step, the scattering curves do not show dramatic changes, and after that, crystal growth is observed. The phase is viscous, which may indicate branching and interconnectivity of the ribbons. The connection of the ribbons in a continuous network should facilitate the delivery of protein molecules to the nucleation site and crystal growth.

### Formation of phase 500–700 Å

It is also possible that the ribbons line up in an orderly package. Therefore, the noncrystalline peaks appearing in the scattering curve can be interpreted as a short-range order between the ribbons. So at the ribbon-like stage, the temporary peaks were observed in a small-angle region of the curves (*q* < 0.025 Å^−1^). These peaks correspond to the spacing parameters of 270–350 Å and 500–700 Å for two directions of ordering. There are two possible interpretations of what this “500–700 Å” phase is. The first interpretation is that these peaks correspond to the lattice parameters 270–350 Å and 500–700 Å and can be associated with the formation of a cholesteric phase made up of ribbon-like structures. The assumption is based on the fact that the lattice parameter is much larger than the ribbon length (see Table S3). In terms of the cholesteric phase, the lattice parameters 500–700 Å correspond to the rotation period along the director axis (the distance over which a full rotation of 360° is completed) known as the pitch^95^. The second peak with a lattice parameter of 270–350 Å corresponds to the length of the ribbons obtained from the approximation of the SAXS curves (see Table S3 and Fig. 2F), which can indicate the additional orientation of the ribbons within this hypothetic cholesteric layers. The second interpretation is that these peaks with lattice parameters of 270–350 Å and 500–700 Å can be associated with the formation of a smectic made up of ribbon-like structures. Smectics are characterized by two order parameters—between the layers and between the elements in one layer. In our case, the smectic layers could be formed by the ribbons. The first smectic peak corresponds to a distance of 500–700 Å between the layers. The second peak corresponds to the distance between the ribbons in one layer of 270–350 Å. The formation of the nematic structures and the orientationally ordered worm-like micelles in the DMPC/DHPC mixtures was shown using polarized optical microscopy and small angle neutron scattering (SANS)^28,75,89^. The reference^28^ related to the pure DMPC/DHPC mixture indicates the appearance of a broad peak at *q* ~ 0.015 Å^−1^ in SANS curves. It corresponds to about 450 Å, which is close to our parameters. Also, the formation of the orientationally ordered worm-like micelles in the DMPC/DHPC mixtures was shown by different methods^28,31,73,75,89,90^.

Unambiguous interpretation of these peaks 270–350 Å and 500–700 Å is impossible by using only SANS and SAXS. We determined that ribbon-like structures are the dominant component of the crystallization system; however, it remains unclear how the ribbons are connected and exactly how they are mutually oriented. To understand the important data of the phase, additional experiments, in particular, a complementary electron microscopy study are desirable. Independently on the true nature of these peaks, we speculated that the formation of such ordered structures might induce protein ordering in the crystallization matrix and promote protein crystallization.

### The lamellar phase Lα

Then we observed a multilamellar phase with a distance parameter of 73 Å. The appearance and growth of crystals were detected by SAXS and vis-microscopy in the steps when the multilamellar phase Lα was also forming in the matrix. This evidence highlights that the presence of extended lamellar structures is an important condition for crystal growth. Presumably, this phase accompanies crystal growth similar to that of observed with LCP grown crystals. The ribbon-like structures are assumed to be the dominant component of the system since the SAXS curves present a good approximation by a form-factor of the ribbons.

### The lamellar phase presumed to be associated with the crystal surface (L_cryst_)

It appears that the presence of a local lamellar (L_cryst_) phase bound with the crystal surface enables the crystal growth. The presence of L_cryst_ is manifested by the appearance of diffraction peaks in our scattering curves. At crystal nucleation, the L_cryst_ has a distance of 68 Å. Then, during the formation and growth of the crystal, this parameter decreases to 64 Å and maintains this value until the end of the observation (60 days). The protein may diffuse to the crystal surface via this phase.

### Summary

Thus, in contrast to the existing paradigm, our study shows that the jelly-like state of the ‘bicelle’ crystallization matrix, rather than the initial bicelle, is the state where crystals grow. We assume that these lamellar structures should be interconnected to help proteins migrate from bilayers to the place of the crystal formation which is necessary conditions for the growth of crystals. Further work to obtain evidence to support this hypothesis is planned.

Importantly, upon crystal formation, a small amount of multilamellar phase appears, and its volume increases concomitantly with volume and the number of the growing crystals. We therefore conclude that the lamellar phase surrounds the crystals and is critical for crystal growth as is also common to LCP crystallization^17,91–93^.

**Figure 7:**
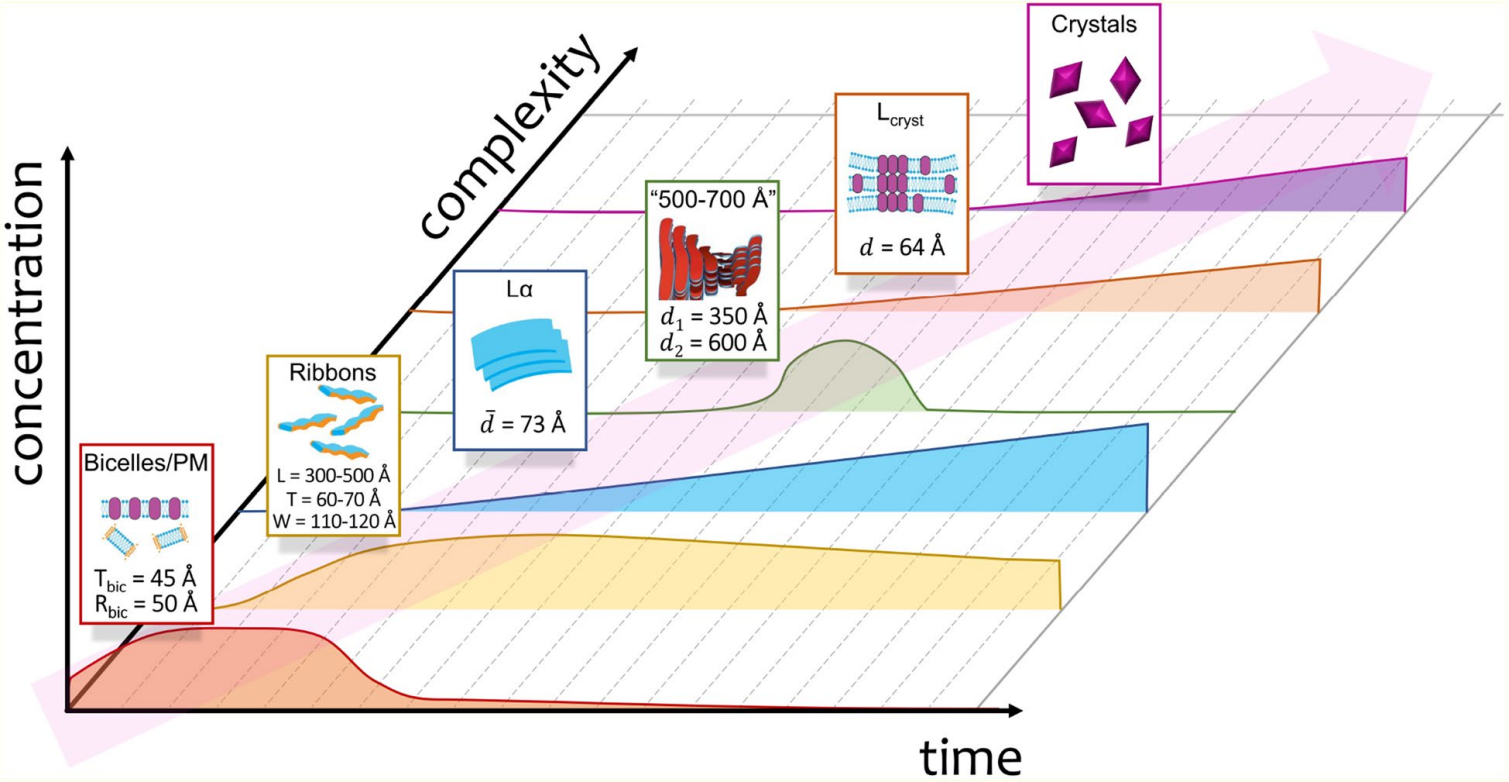
The scheme demonstrating the evolution of the crystallization matrix and sequential appearance/disappearance of various structural elements: a mixture of bicelles and PMs, ribbons, the lamellar phase Lα, the “phase 500–700 Å”, the local lamellar phase L_cryst_ and BR crystals (see more extended description in the main text). Following Katsaras et al.^31^, we present the “phase 500–700 Å” as chiral nematic; however, the true nature of this phase is still unclear. The axis correspond to time, complexity (i.e. number/quantity of the new appeared structural element), and concentration, respectively. Concentration is given in arbitrary units (structural elements have different concentration ranges; here the concentration is presented on the same scale for clarity; dependencies of concentrations vs. time are shown qualitatively).

We summarize all available information about the evolution of the crystallization matrix that has been mentioned in our work in the following scheme of sequential appearance/disappearance of various structural elements shown in Fig. 7. The process starts with a fluid phase containing a simple mixture of bicelles and purple membranes. Initially, the concentration of bicelles and PMs increases due to a decrease in volume of the crystallization matrix upon drying (see Fig. S4). Then bicelles fuse to ribbons; this process is accompanied by the PM dissolution. Ribbons form a gelly-like phase, which is the main component of the crystallization matrix during the appearance and growth of BR crystals. However, between the appearance of ribbons and crystals, several more types of structural elements appear: the lamellar lipid phase Lα, the “phase 500–700 Å” and the local lamellar phase L_cryst_. Lα corresponds to multilamellar lipid membranes appearing due to the fusion of ribbons. The amount of Lα increases (see Fig. S5A) simultaneously with a slow decrease of ribbon concentration. For a time much longer than crystals grew (~ 100 days), ribbons can completely transform into the Lα phase (see Fig. 2G). After Lα, the “phase 500–700 Å” was detected. This high-ordered structure has a lattice parameter equal to or even higher than the length of ribbons. According to the literature^28,31,73,75,89,90^, such structures can correspond to smectics or cholesterics (chiral nematics). The exact role of this high-ordered structure for the protein crystallization process is questionable. The “phase 500–700 Å” appears for about a week and then it disappears. L_cryst_ corresponds to multilayer membranes located on the surface of protein crystals and allowing the protein to diffuse to the crystal surface. In those samples where crystal growth was observed, the appearance of L_cryst_ precedes the appearance of crystals, which indicates that L_cryst_ also corresponds to the protein nucleation zones. Then the intensity of the I(q) peaks from L_cryst_ and from crystals grow synchronously (Fig. S5(A)). Finally, membrane protein crystals appear in the crystallization sample.

Our results help to shed more light on *in meso* MP crystallization and while questions remain the results reported here strongly support the use of this type of crystallization using rational design, making it considerably more efficient. This approach might also help with efficient crystallization of MPs for structure-based drug design, development of vaccines based on MPs belonging to different pathogens e.g. SARS-CoV-2, and other biomedical applications.

## Materials and methods

### Materials

Lipid 1,2-dimyristoyl-sn-glycero-3-phosphocholine (DMPC) was purchased from Avanti Polar Lipids Inc; 3-([3-Cholamidopropyl]dimethylammonio)-2-hydroxy-1-propanesulfonate (CHAPSO), 2,5-Hexanediol, triethylene glycol were purchased from Sigma-Aldrich.

### Preparation of the crystallization system

Protein/bicelle mixture was prepared according to the procedure described previously^96^. CHAPSO and DMPC were mixed at a molar ratio of 1:2.69 (CHAPSO:DMPC). The deionized water (18.2 MΩ·cm) was added to this mixture to achieve a final concentration of bicelles of 35% (w/v). The mixture was homogenized through cyclic cooling on ice, vortexing, and brief heating (at 40 °C). The prepared mixture was stored at −20 °C.

Purple membranes containing bacteriorhodopsin (BR) were purified from *Halobium Salinarum* cells, as described in^97^, and concentrated to a BR concentration of ~ 10 mg/ml. Cooled purple membranes and 35% solution of bicelles were mixed in a ratio of 4:1 (v/v), gently resuspended, and placed on ice. The precipitant solution was added to the membrane/bicelles mixture in a ratio of 1:4 (v/v) so that the buffer composition of the protein/bicelle mixture was 0.49 M NaH_2_PO_4_, 36 mM hexanediol, 1.26% triethylene glycol. The precipitant solution contained 2.45 M NaH_2_PO_4_, 180 mM hexanediol, 3.5% triethylene glycol, pH = 3.7. The final protein-bicelle solution was incubated on ice for 30 min and placed into capillaries. The final protein/bicelle mixture contained 5.6% bicelles (total concentration DMPC + CHAPSO) and 8–9 mg/ml BR (we studied several series of capillaries).

### Crystallization set-up in capillaries

Since standard crystallization tools (such as sitting drop or hanging drop) are not suitable for simultaneous performing small angle experiments, we developed an equivalent crystallization procedure in glass capillaries.

The scheme of a capillary is presented in Figure S1. Borosilicate glass capillaries with an outside diameter of 1.5 mm and a wall thickness of 0.01 mm were used; the length of the capillaries was 80 mm. The transfer of the crystallization system (BR/bicelle mixture) into the capillaries was performed using a spinal needle with an outer diameter of 1.1 mm and a length of 90 mm. Before filling the capillary, all the materials (capillaries, needles, and solutions) were cooled to ensure liquid consistency of the protein-bicelle suspension.

The protein-bicelle suspension was placed at the bottom of the capillary. The precipitant solution was placed at the top so that the air gap of 6–8 mm formed between the protein-bicelle suspension and the precipitant solution. The end of the capillary was sealed with wax (Figure S2 and Fig. 1).

One batch of the capillaries was stored at 32 °C, the other one – at room temperature. We noticed that changes in the crystallization system were very slow at room temperature due to a small evaporation area that was limited by the diameter of the capillary. Therefore, after 4 weeks we placed all the capillaries in an incubator box at 32 °C. The crystallization was done at 32 °C. The crystal growth was monitored by a vis-microscope.

### SAXS measurements and data reduction

Most SAXS experiments were performed at a Rigaku instrument at the Moscow Institute of Physics and Technology (Dolgoprudny, Russia)^98^. The Rigaku SAXS instrument was equipped with a pinhole camera attached to a rotating anode X-ray high-flux beam generator (MicroMax 007-HF) operating at 40 kV and 30 mA (1200 W). The X-ray wavelength λ was 1.54 Å. A multiwire gas-filled detector Rigaku ASM DTR Triton 200 (diameter of the active area is 200 mm, pixel size is ~ 260 μm) was placed at a distance of 2.0 m from the samples (the covered *q*-range is 0.006–0.19 Å^−1^) and/or 0.5 m (0.024 Å^−1^ ≤ *q* ≤ 0.8 Å^−1^). The azimuthal integration of the obtained 2D images was performed using the Saxsgui software (Rigaku Innovative Technologies, Inc., and JJ X-ray System Aps). An additional SAXS experiment for characterizing unit cells of BR crystals in one sample was performed at the bioSAXS beamline BM-29 at the ESRF (Grenoble, France)^99^. The working energy of BM-29 (ESRF) was 12.5 keV, the experimental hutch was equipped with a marble table housing a modular-length flight tube (3.5 m sample to detector distance used for this experiment), a 2D detector (Pilatus 1 M), and an automated sample changer, and the achievable *q* value range was 0.0025–0.5 Å^−1^. The data collection, processing, and initial analysis were performed in an automated manner using BsxCuBE and the dedicated beamline software within the EDNA framework^100^.

### SANS measurement and data reduction

SANS measurements were performed at the YuMO time of a flight spectrometer (IBR-2, Dubna, Russia) with a two-detector system^101,102^. The detectors’ positions were 4.5 and 13 m from the sample positions. The used neutron wavelengths *λ* from 0.5 to 8 Å with the achievable *q*-range from 0.007–0.5 Å^−1^. Raw data treatment was processed with the SAS program^103^.

### Calculation of unit-cell dimensions for BR crystals

For the recalculation of the unit-cell dimensions from SAXS data, the following sum of squares of the related discrepancies was minimized:

1 $$\sum_{i}{\left(\frac{{q}_{exp}^{i}-{q}_{theor}\left({\left[h k l\right]}_{i}\right)}{{q}_{theor}\left({\left[h k l\right]}_{i}\right)}\right)}^{2}\to min,$$

where *q*_*exp*_^*i*^ is the position of the *i*th experimental peak, *q*_*theor*_ ([*hkl*]_*i*_) is the theoretical peak position that corresponds to the Miller indexes [*hkl*]_*i*_ giving the closest peak position to *q*_*exp*_^*i*^ (see Table S4). The theoretical peak positions for a crystal with a monoclinic unit cell are given by the following expression (2):

2 $${q}_{theor}\left(\left[h k l\right]\right)=\frac{2 \pi }{\mathrm{sin}\beta } \sqrt{{\left(\frac{h}{a}\right)}^{2}+{\left(\frac{k\ \mathrm{sin}\beta }{b}\right)}^{2}+{\left(\frac{l}{c}\right)}^{2}-\frac{2\ h\ l\ \mathrm{cos}\beta }{a c}.}$$

### SAS data analysis and modeling

The Primus program from the ATSAS software suite^104^ was used for the primary processing of SAXS and SANS 1D profiles *I(q)*. SAXS data for water solutions of amphiphilic molecules (unilamellar liposomes, bicelles, nanodiscs, detergent micelles and solubilized membrane proteins) typically demonstrate the intensity maximum in the range 0.1–0.2 Å^-1^ (see, for example, Fig. 2A), which is caused by strong inhomogeneities in electron density (hydrophobic parts usually have a negative contrast relative to the solvent, while hydrophilic parts have a positive contrast). In studies of such systems by SAXS, errors and ambiguities can arise if using models with homogeneous electron density profiles, which was shown for the membrane protein complex *Np*SRII/*Np*HtrII solubilized in a detergent^105,106^. Therefore, correct interpretation of SAXS data of such systems requires models with different scattering length densities for different parts of the studied objects (in case of bicelles—a detergent belt, hydrophilic heads, and hydrophobic tails of the lipid bilayer). For this purpose, two models were used.

**Figure 8:**
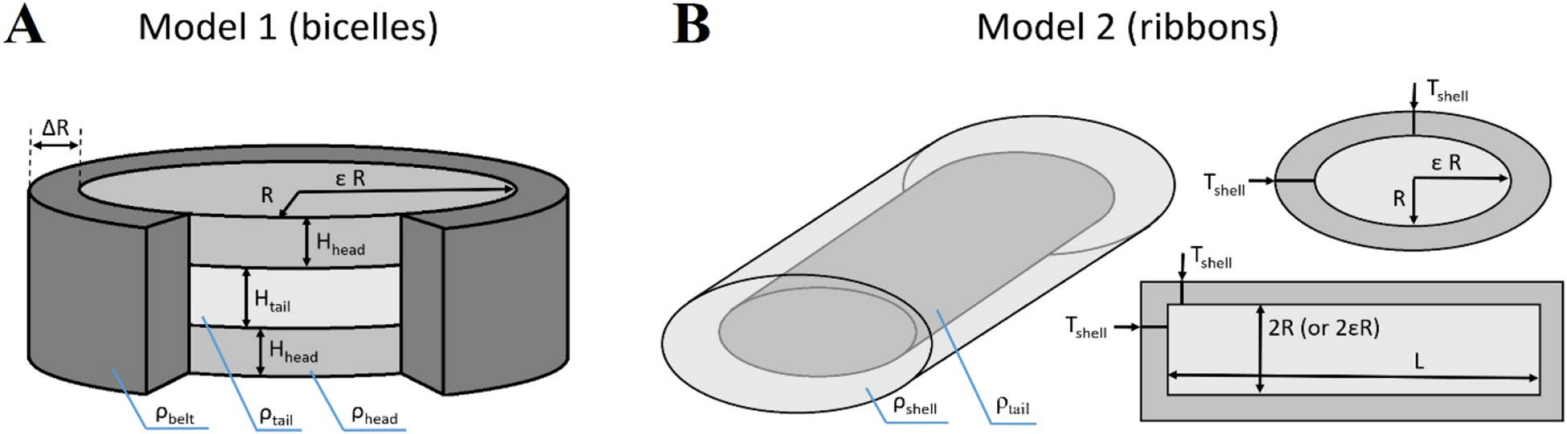
Models used for the approximation of the SAS data. (**A**) Model 1: circular cylinder with a core–shell scattering length density profile (in our calculations, we used *ε* = 1 for the bicelles). (**B**) Model 2: elliptical cylinder with a core–shell scattering length density profile.

Model 1 (used for the bicelles) is a circular cylinder with a core–shell scattering length density profile (see Fig. 8A, we used a plugin model based on the SasView model of the cylinder and the core_shell_bicelle). In accordance with the previously reported data for the 3S model of the DMPC bilayer^107^ (which is analogous to our case), the thickness of this core (*H*_*tail*_) could be fixed at a value of 28.8 Å; in accordance with the known area per lipid, *A*_*L*_ = 61.8 Å^2^, the SLD of the hydrophobic core (*ρ*_*tail*_) could be calculated and fixed (see Table S3). The minor radius of the core cylinder is R, the major/minor radius ratio *ε* = 1. The core faces correspond to the hydrophilic polar heads of DMPC, and the water molecules bound to the polar heads. The thickness of this face layer (*H*_*head*_) is a fitting parameter. We assumed that the belt of the cylinder is formed by CHAPSO. In accordance with the atomic model of the CHAPSO molecule^108^, the belt thickness *ΔR* in our calculations was fixed on the value of 11.4 Å.

Model 2 (used for the ribbons) is an elliptical cylinder with a core–shell scattering length density profile (see Fig. 8B, we used a plugin model based on two SasView models from the “cylinder” category: a core_shell_cylinder and an elliptical_cylinder).

The SAS curves fitting with the models of bicelles and ribbons was done using the SasView 4.2.2 program^109^. The optimization of the structural parameters in the mentioned models was done by minimization of the following expression:

3 $${\chi }^{2}=\frac{1}{{N}_{exp}-{N}_{param}}\sum_{i=1}^{{N}_{exp}}{\left(\frac{{I}_{i}-{I}_{model}\left({q}_{i}\right)}{{\sigma }_{i}}\right)}^{2},$$

where *N*_*exp*_ and *N*_*param*_ are the numbers of the experimental points and the fitted parameters, respectively; *(q*_*i*_,* I*_*i*_*, σ*_*i*_*)* is a set of the SAS experimental data. Equations (4–7) for the model curve *I*_*model*_*(q*_*i*_*)* are given in the Text Document S1.

The calculations were carried out on the assumption that the structure factor can be neglected (*S(q)* = 1). Even though the sample concentrations are high (5.6–14%), and some influence of the structure factor on the scattering curve is inevitable, this influence is not so critical to determine the type of the object, in particular, to distinguish between the bicelles and the ribbons, which was demonstrated in work (*19*). However, the structural parameters determined during the fitting process may differ from the real ones. In fact, there is no way to take *S(q)* into account correctly as there are no appropriate theoretical models. It is a big challenge to consider the effect of the structure factor at lower concentrations by using dilution and following the SAXS experiments since the structure of the studied objects depends on concentration and changes with time. Thus, experiments with other sample concentrations will not allow obtaining information about those states of the system that occur in real experiments of membrane protein crystallization and that are studied in this work.

## Supplementary Information

Supplementary Information 1.

Supplementary Information 2.

## Data Availability

Data supporting the findings of this manuscript are available from the corresponding author upon reasonable request.
