# Supplementary material for: Mechanisms of membrane protein crystallization in ‘bicelles’
Source: Sci Rep. 2022 Jun 30;12:11109. doi: 10.1038/s41598-022-13945-0 (PMC9246360; doi:10.1038/s41598-022-13945-0)
Supplement: Supplementary file 1 — Supplementary Information 1. [file 41598_2022_13945_MOESM1_ESM.pdf]

Supplementary Information for
Mechanisms of membrane protein crystallization in 'bicelles'

Tatiana N. Murugova[1,2,†,*], Oleksandr I. Ivankov[1,3,4,†], Yury L. Ryzhykau[2,1,†], Dmytro V. Soloviov[1,2,4], Kirill V. Kovalev[5], Daria V. Skachkova[1], Adam Round[6,7], Christian Baeken[8,9], Andrii V. Ishchenko[2], Oleksandr A. Volkov[8,9], Andrey V. Rogachev[1,2], Alexey V. Vlasov[2,1], Alexander I. Kuklin[1,2,*], Valentin I. Gordeliy[10,*]

[1] Frank Laboratory of Neutron Physics, Joint Institute for Nuclear Research, 141980 Dubna, Russia
[2] Research Center for Mechanisms of Aging and Age-Related Diseases, Moscow Institute of Physics and Technology, 141700 Dolgoprudny, Russia
[3] Taras Shevchenko National University, 01033 Kyiv, Ukraine
[4] Institute for Safety Problems of Nuclear Power Plants of the Ukrainian NAS, 03028 Kyiv, Ukraine
[5] EMBL Hamburg outstation, 22607 Hamburg, Germany
[6] Previously at EMBL-Grenoble outstation, 38000 Grenoble, France
[7] Single Particles, Clusters, and Biomolecules and Serial Femtosecond Crystallography (SPB/SFX) Instrument, European XFEL GmbH, 22869 Schenefeld, Germany
[8] Institute of Biological Information Processing (IBI-7: Structural Biochemistry), Forschungszentrum Jülich, 52425 Jülich, Germany
[9] JuStruct: Jülich Center for Structural Biology, Forschungszentrum Jülich, 52428 Jülich, Germany
[10] Institut de Biologie Structurale Jean-Pierre Ebel, Université Grenoble Alpes–Commissariat à l'Energie Atomique et aux Energies Alternatives–CNRS, F-38027 Grenoble, France

* Corresponding authors. Email: murugova@jinr.ru (TNM); kuklin@nf.jinr.ru (AIK); valentin.gordeliy@ibs.fr (VIG).

† These authors contributed equally to this work.

This PDF file includes:

Figures S1 to S6
Legend for Table S1
Tables S2 to S4
Supplementary Text Document S1

Other supplementary materials for this manuscript include the following:

Table S1 (separate file)

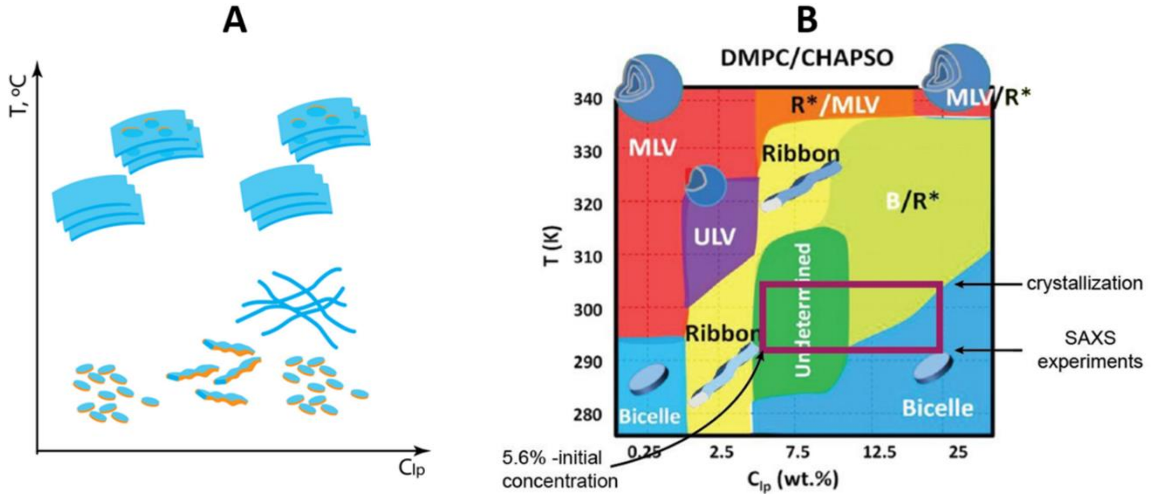

Fig. S1. Phase diagram for the DMPC/CHAPSO bicellar system. (**A**) – Schematic phase diagram showing structural behavior of the bicellar systems. (**B**) – The phase diagram for the DMPC/CHAPSO mixture at molar ratio Q = 3. The purple rectangle highlights the conditions used in this work. Panel B was adapted with permission from (19). Copyright (2013) American Chemical Society.

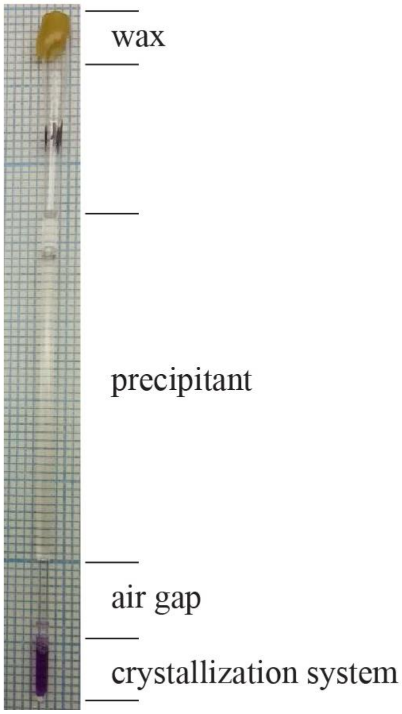

Fig. S2. Representative photograph of the capillary with the crystallization system and the precipitant.

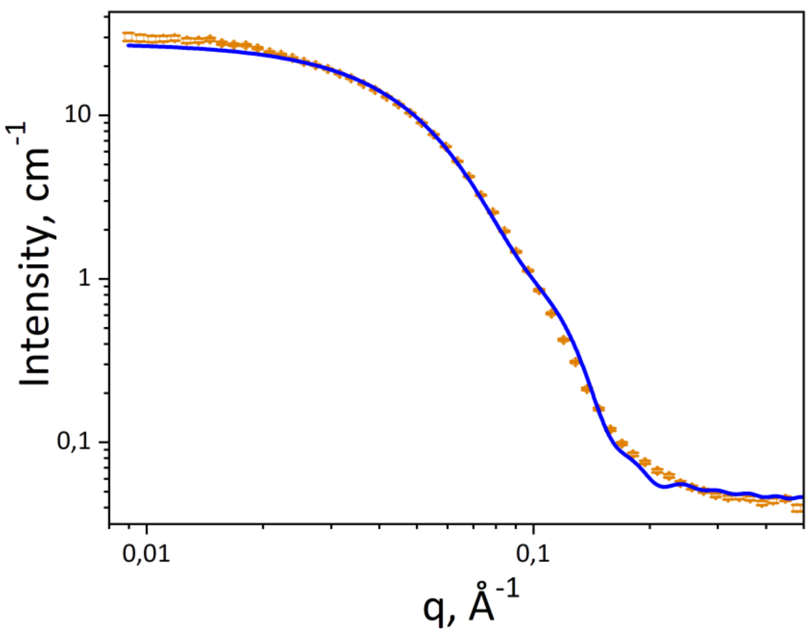

Fig. S3. SANS data for the bicelles. Experimental SANS data for bicelles in D_2O–buffer (orange hollow circles) and corresponding fit by the form-factor of bicelles (blue curve, see fit parameters in Table 2).

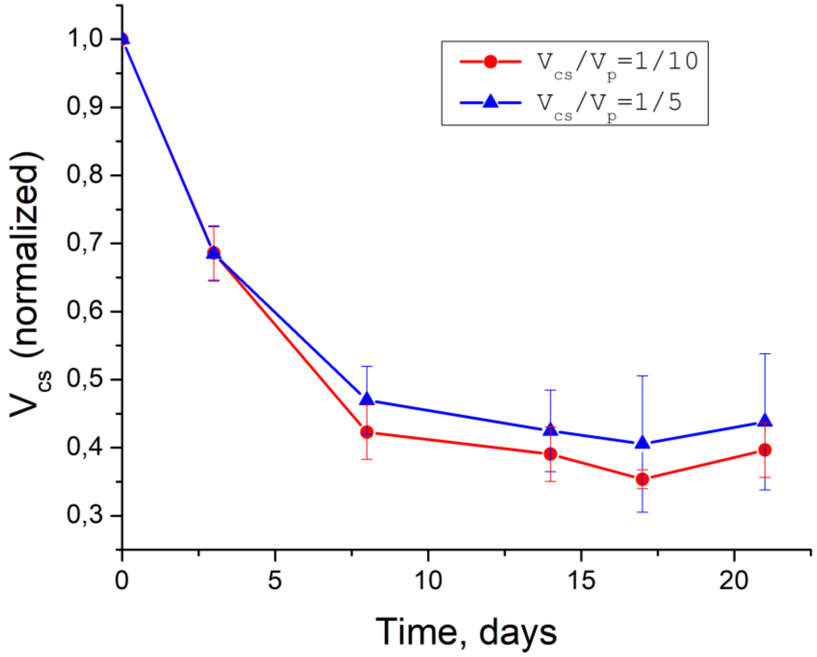

Fig. S4. Changes in the volume of the crystallization system during the evaporation process. The observed height of the crystallization system in a capillary during the crystallization process. The presented values are averaged over 4-5 capillaries and normalized on the height of the system on the first day of the pouring system in a capillary. V_{cs}/V_p – volume ratio of the crystallization system and the precipitant in the capillary (V_{cs} and V_p mean volume of the crystallization system and the precipitant, correspondingly).

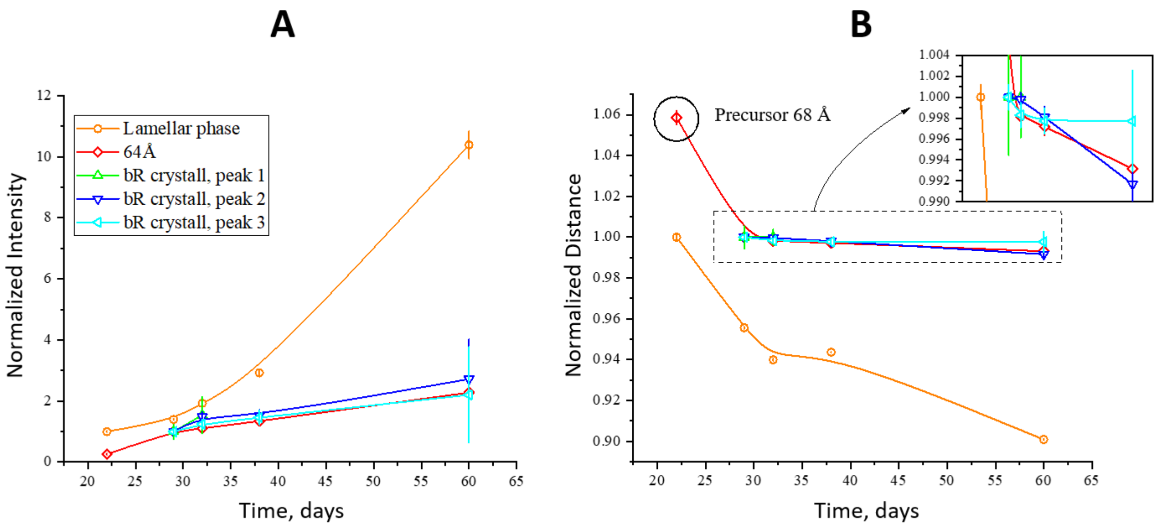

Fig. S5. Time dependence for the intensities and the positions of the diffraction peaks. (**A**) –Time dependence for the intensities of the diffraction peaks for the lamellar phase Lα (1st order peak, orange circles), the local lamellar phase L_{cryst} (red rhombus), and the BR crystals (green, blue and cyan triangles). Since during crystallization, the scattering intensities changed with concentration, the scattering curves were scaled to make baselines overlap, then the baseline was subtracted. (**B**) – Time dependence for the positions of the diffraction peaks for the lamellar phase Lα (1st order peak), the local lamellar phase L_{cryst}, and the BR crystals (with the same designations as in part A). The peak positions are transformed into distances as $2\pi / q$. The peak position and the intensities were calculated from Gaussian approximation; the obtained values were normalized on the values for the first day of observing the corresponding peak.

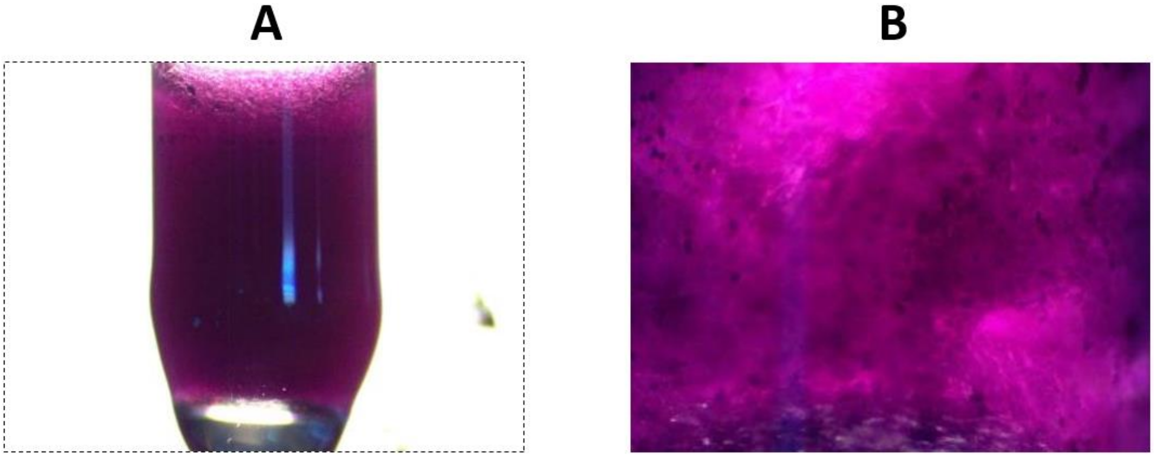

Fig. S6. Photograph of the crystallization phase for the sample that did not induce the appearance of the peaks from the BR crystals but show the diffraction peak corresponding to 68 Å. (**A**) – General view of a part of the capillary with the sample. (**B**) – Detailed micrograph of the sample.

Table S1. (Separate file) The list of the membrane proteins whose structure was resolved by X-ray crystallography using bicellar systems.

Table S2. Approximation parameters of the bicelles. The parameters of the SAXS and SANS curves for the pure DMPC/CHAPSO bicellar mixtures (without PMs) and for the difference scattering intensity for the crystallization system in steps 2 and 3 by a form-factor of the bicelles (see equations [5-7] in the Text Document S1). The corresponding theoretical curves are presented in Fig. 2(A-D) and Fig. S3. Parameter designations are given in "Materials and Methods", see section "SAS data analysis and modeling".

Experiment	SAXS				SANS
	Pure bicellar mixture in step 2	Pure bicellar mixture in step 3	Difference scattering intensity for the crystallization system in step 2	Difference scattering intensity for the crystallization system in step 3	Pure bicellar mixture in step 2
SAS curve	Fig. 2(A)	Fig. 2(C)	Fig. 2(B)	Fig. 2(D)	Fig. S3
R (Å)	49.5 ± 1.5	39.6 ± 0.3	38.8 ± 0.3	37.3 ± 0.3	37.7 ± 0.2
ΔR (Å)	11.4 (fixed)				
H_{tail} (Å)	28.8 (fixed)				
H_{head} (Å)	8.15 ± 0.15	8.15 (fixed)*			
ρ_{belt} †	0.372 ± 0.002	0.3907 ± 0.0008	0.3836 ± 0.0007	0.3822 ± 0.0007	2.84 ± 0.10
ρ_{tail} †	0.23598 (fixed)				-0.327 (fixed)
ρ_{head} (e/Å^3)†	0.544 ± 0.003	0.4812 ± 0.0008	0.544 (fixed)	0.4705 ± 0.0007	5.400 (fixed)
ρ_{buffer} (e/Å^3)†	0.3459 (fixed)	0.3634 (fixed)	0.3459 (fixed)	0.3634 (fixed)	6.356 (fixed)
χ^2	1.622	1.312	1.034	1.131	33.24

* – this parameter was obtained from the first fit of the curve for the pure bicellar mixture in step 2. The same value was used in the following fits to decrease the number of free parameters.

† – units of the scattering length density (SLD) are e/Å^3 and 10^{-6} Å^{-2} for SAXS and SANS, respectively.

Table S3. Approximation parameters of the ribbons. The parameters of the SAXS curves approximation for the pure DMPC/CHAPSO mixtures in Step 3 and the crystallization system (DMPC/CHAPSO/PM) in steps 3 and 4 by a form-factor of the ribbons (see equations [5-7] in the Supplementary Text Document S1). The corresponding theoretical curves are presented in Fig. 2(E, F, and H). Parameter designations are given in "Materials and Methods", see section "SAS data analysis and modeling".

	DMPC/CHAPSO mixture (no PM)		Crystallization system DMPC/CHAPSO/PM	
SAXS curve	Fig. 2(E)		Fig. 2(F)	Fig. 2(H)
Fraction of the ribbons* (%)	33.3 ± 0.2	100 (fixed)	100 (fixed) †	100 (fixed) †
R (Å)	24.64 ± 0.07	19.91 ± 0.05	21.76 ± 0.09	22.58 ± 0.05
ε	1.908 ± 0.011	2.142 ± 0.009	2.07 ± 0.02	2.104 ± 0.016
T_{shell} (Å)	11.4 (fixed)			
L (Å)	334 ± 2	331 ± 2	322 ± 3	552 ± 5
ρ_{shell} (e/Å^3)	0.4537 ± 0.0006	0.4408 ± 0.0004	0.4411 ± 0.0008	0.4555 ± 0.0006
ρ_{tail} (e/Å^3)	0.23598 (fixed)		0.246805 (fixed)	
ρ_{buffer} (e/Å^3)	0.3634 (fixed)			
χ^2	1.229	3.508	1.500	2.035

* – volume fraction of the ribbons in relative to the volume of bicelles and the ribbons together.

† – the possible presence of the bicelles does not significantly change χ^2 (data not shown).

Table S4. Diffraction on the BR crystals. First and second columns – positions and integral widths of experimentally observed diffraction peaks (see Fig. 5), correspondingly. Third column – the Miller indexes (for the BR crystals) or reflex numbers (for Lα or L_{cryst}) corresponding to the observed peaks. Fourth and fifth columns – theoretical peak positions (see equation [2]) corresponding to the Miller indexes shown in the third column and the unit-cell dimensions from the previously reported data (ref. (7) in the main text) and our work, correspondingly.

$q_{exp}{}^{i}$ (Å^{-1})	$\Delta q_{int}{}^{i}$ (Å^{-1})	$[h\,k\,l\,]_i$	q_{theor} ($[h\,k\,l\,]_i$) (Å^{-1}) (7)	q_{theor} ($[h\,k\,l\,]_i$) (Å^{-1}) (this work)
0.08210	0.002476	Lα [reflex 1]	–	–
0.09764	0.002063	L_{cryst} [reflex 1]	–	–
0.12163	0.001502	[0 0 1]	0.12264	0.12178
0.14856	0.001940	[1 0 0]	0.15234	0.14785
0.15696	0.008347	[1 1 0]	0.16291	0.15862
0.16417	0.003806	Lα [reflex 2]	–	–
0.17227	0.001723	[0 3 0]	0.17309	0.17239
0.17748	0.002732	[1 1 -1]	0.16319	0.17579
0.18951	0.002125	[1 2 0]	0.19112	0.18726
0.19465	0.003935	L_{cryst} [reflex 2]	–	–
0.19934	0.001184	[1 2 -1]	0.19136	0.20201
0.20864	0.003685	[0 3 1]	0.21214	0.21107
0.22710	0.003234	[1 3 0]	0.23059	0.22711
0.23155	0.005038	[0 4 0]	0.23079	0.22986
0.24492	0.005640	Lα [reflex 3]	–	–
0.27040	0.003434	[0 2 2]	0.27107	0.26932
0.28888	0.006793	L_{cryst} [reflex 3]	–	–
0.29951	0.005389	[0 3 2]	0.30021	0.29840
0.31555	0.006292	[1 0 2]	0.33656	0.31522
0.32618	0.006216	Lα [reflex 4]	–	–
0.33471	0.005640	[0 3 2]	0.30021	0.33490
0.34597	0.003459	[2 0 1]	0.37118	0.34705

Supplementary Text Document S1

The model function used to approximate the experimental SAXS and SANS intensity profiles is given by the following expression:

$$I_{model}(q) = n\, S(q)\, P(q) + bkg, \qquad [4]$$

where *n* is the number density of the particles, *P(q)* is the theoretical intensity for an isolated particle, and *S(q)* is the structure factor accounting for inter-particle interaction effects. The additional constant *bkg* accounts for possible systematic errors due to mismatched buffers in the experimental data. For the model of the elliptical cylinder with a core-shell scattering length density profile (shown in Fig. 8(A)), *P(q)* is given by the following expression:

$$P(q) = \int_0^{\pi} d\Theta \int_0^{2\pi} |F(q,\theta,\varphi)|^2 d\varphi \qquad [5]$$

where

$$F(q,\theta,\varphi) = (\rho_{tail} - \rho_{head})\, \pi\varepsilon R^2 \frac{2J_1(qR'(\varphi)sin\theta)}{qR'(\varphi)sin\theta} \frac{sin(qH_{tail}\, cos\theta/2)}{q\, cos\theta/2} +$$

$$+(\rho_{head} - \rho_{belt})\, \pi\varepsilon R^2 \frac{2J_1(qR'(\varphi)sin\theta)}{qR'(\varphi)sin\theta} \frac{sin(q(H_{tail} + 2H_{head})cos\theta/2)}{q\, cos\theta/2} + \qquad [6]$$

$$+\left(\rho_{belt} - \rho_{buffer}\right) \pi\varepsilon\Delta R(2R + \Delta R) \times$$

$$\times \frac{2J_1(q(R'(\varphi) + \Delta R)sin\theta)}{q(R'(\varphi) + \Delta R)sin\theta} \frac{sin(q(H_{tail} + 2H_{head})cos\theta/2)}{q\, cos\theta/2},$$

where the effective radius *R'(φ)* is equal to

$$R'(\varphi) = \frac{R}{\sqrt{2}}\sqrt{(1+\varepsilon^2) + (1-\varepsilon^2)\, cos\varphi}. \qquad [7]$$

Theoretical *P(q)* for the bicelles and the ribbons (see Fig. 8) are special cases of equations [5-7] corresponding to conditions $\varepsilon = 1$ (for Model 1) and $T_{shell} = H_{head} = \Delta R$, $L = H_{tail}$, and $\rho_{shell} = \rho_{belt} = \rho_{head}$ (for Model 2), respectively.

Supplementary Text Document S2

SAXS profiles for bicelles. The SAXS profiles presented in the mentioned works (ref. (34, 77-79) in the main text) also show a secondary peak at q ~ 0.1-0.2 $Å^{-1}$, typical for bicelles which we also observed in our experiments. The DMPC molecules consist of two principally different parts: hydrophobic tails ($C_{26}H_{54}$, the neutron scattering length is b_n = – 29.11 fm, the number of electrons is N_e = 210) and hydrophilic heads ($C_{10}H_{18}O_8PN$, b_n = 60.072 fm, N_e = 164). The hydrophobic core of the bicells corresponds to the hydrophobic tails of the DMPC molecules and has a negative contrast of electron density with the solvent. The mentioned secondary peak at q ~ 0.1-0.2 $Å^{-1}$ is a typical one for these core-shell systems.

Differences in bicelle radii. The value of a bicell's radius R obtained by fitting SAS curves from different samples varies (see Table S2), which is not surprising since the bicelles for these measurements were prepared independently, and the exact repeatability of the values of the structural parameters cannot be guaranteed precisely.

Fitting of difference intensities. The data sets corresponding to the mixture of the bicelles with PMs at steps 1-2 and at the beginning of step 3 could be described as sum of scattering data from PMs and pure bicelles. To prove it, we calculated difference intensity profiles that were calculated as the result of subtracting the SAXS curve for pure PMs from the SAXS curves from the DMPC/CHAPSO/PMs mixture. Then, were approximated these difference intensities in accordance with Model 1; the parameters of the DMPC bilayers were fixed on the values previously calculated for the "pure" bicellar mixture. The coefficients for the subtraction of the curves accounting for scattering from PMs (see Figure 2(B, D)) were also fit parameters.

Buffer SLD values at different steps. For the initial mixture at steps 1-2, the scattering length density of the solvent ρ_{buffer} is calculated in accordance with the buffer composition (0.3459 e/$Å^3$ for the 0.5 M Na_2HPO_4; neutron SLD is 6.356×10^{-6} $Å^{-2}$). For steps 3 and 4, the SLD of the solvent will be higher due to an increase of salt concentration during the evaporation process. For calculating this parameter, we were guided by the changes in the volume of the crystallization system described in Figure S4: assuming a decrease of the volume to 40% from the starting point, the final Na_2HPO_4 concentration is 1.25 M, which corresponds to a buffer electron density of 0.3634 e/$Å^3$.

SLD changes in bicelles. A decrease of the ρ_{head} parameter from 0.544 to 0.4712 e/$Å^3$ (see Table S2) corresponds to a decrease of the average number of water molecules per DMPC hydrophilic head from 11.0 to 7.8, caused by the evaporation process, in which salt concentration becomes higher, and water activity decreases.

SLD changes in ribbons. For the ribbons formed in the pure DMPC/CHAPSO mixture, we approximate ρ_{core} at the value of ρ_{tail} for the bicelles (see Table S2). In the case of DMPC/CHAPSO/PMs mixture, we assumed that BR molecules incorporated directly into ribbons after PM dissociation. Therefore, taking into account the cross-section area ratio of BR to DMPC in a membrane of ~1:17, we estimate the density of the inner part of the ribbons by the formula $\rho_{tail}^{ribbons\ with\ BR} = (1/18)\cdot\rho_{prot} + (17/18)\cdot\rho_{tail}^{pure\ ribbons}$, where the electron density of membrane protein is ρ_{prot} = 0.42 e/$Å^3$. In both cases of DMPC/CHAPSO mixture with/without PMs, ρ_{shell} was a fitted parameter. Its values obtained by fitting SAXS data are in the range from 0.44 to 0.46 e/$Å^3$ (see Table S3).
